# Disruption of both chloroplastic and cytosolic FBPase genes results in a dwarf phenotype and important starch and metabolite changes in *Arabidopsis thaliana*

**DOI:** 10.1093/jxb/erv062

**Published:** 2015-03-05

**Authors:** José A. Rojas-González, Mauricio Soto-Súarez, Ángel García-Díaz, María C. Romero-Puertas, Luisa M. Sandalio, Ángel Mérida, Ina Thormählen, Peter Geigenberger, Antonio J. Serrato, Mariam Sahrawy

**Affiliations:** ^1^Departamento de Bioquímica, Biología Molecular y Celular de Plantas, Estación Experimental del Zaidín, Consejo Superior de Investigaciones Científicas, C/Profesor Albareda 1, 18008, Granada, Spain; ^2^Instituto de Bioquímica Vegetal y Fotosíntesis, CSIC-US, Avda Américo Vespucio, 49, 41092, Sevilla, Spain; ^3^Ludwig Maximilians University of Munich, Biology Department I, Plant Metabolism, Grosshaderner Str. 2–4, D-82152 Planegg, Germany

**Keywords:** Chloroplastic, cytosolic, fructose-1, 6-bisphosphatase, knockout mutants, metabolites, starch, sucrose.

## Abstract

In this study, evidence is provided for the role of fructose-1,6-bisphosphatases (FBPases) in plant development and carbohydrate synthesis and distribution by analysing two *Arabidopsis thaliana* T-DNA knockout mutant lines, *cyfbp* and *cfbp1*, and one double mutant *cyfbp cfbp1* which affect each FBPase isoform, cytosolic and chloroplastic, respectively. cyFBP is involved in sucrose synthesis, whilst cFBP1 is a key enzyme in the Calvin–Benson cycle. In addition to the smaller rosette size and lower rate of photosynthesis, the lack of cFBP1 in the mutants *cfbp1* and *cyfbp cfbp1* leads to a lower content of soluble sugars, less starch accumulation, and a greater superoxide dismutase (SOD) activity. The mutants also had some developmental alterations, including stomatal opening defects and increased numbers of root vascular layers. Complementation also confirmed that the mutant phenotypes were caused by disruption of the *cFBP1* gene. *cyfbp* mutant plants without cyFBP showed a higher starch content in the chloroplasts, but this did not greatly affect the phenotype. Notably, the sucrose content in *cyfbp* was close to that found in the wild type. The *cyfbp cfbp1* double mutant displayed features of both parental lines but had the *cfbp1* phenotype. All the mutants accumulated fructose-1,6-bisphosphate and triose-phosphate during the light period. These results prove that while the lack of cFBP1 induces important changes in a wide range of metabolites such as amino acids, sugars, and organic acids, the lack of cyFBP activity in *Arabidopsis* essentially provokes a carbon metabolism imbalance which does not compromise the viability of the double mutant *cyfbp cfbp1*.

## Highlight

Lack of chloroplastic FBPase induces a dramatic reduction in plant development, while loss of the cytosolic enzyme increases the starch content without affecting the phenotype. Inactivation of both enzymes causes a wide range of metabolite changes.

## Introduction

Sucrose and starch are the major end-products in higher plants, and their functions are essential for plant development (Geigenberger, 2011). The rate of net CO_2_ fixation determines the rate of starch and sucrose synthesis. The photosynthetic carbon reduction cycle (the Calvin–Benson cycle) is responsible for the formation of these carbohydrates after the fixation and reduction of atmospheric CO_2_, the first important intermediate metabolites being the triose-phosphates (TPs). By condensation, TPs form fructose-1,6-bisphosphate (F1,6BP) which is used to synthesize starch in the chloroplast and sucrose in the cytosol. Fructose-1,6-bisphosphatase (FBPase) catalyses the breakdown of F1,6BP to fructose-6-phosphate (F6P) and Pi (Zimmermann *et al.*, 1976). Three FBPases have been described so far in the plant cell, the cytosolic enzyme (cyFBP) which is involved in sucrose synthesis and gluconeogenesis (Cséke and Buchanan, 1986), and two other chloroplastidial isoforms (cFBP1 and cFBP2) (Serrato *et al.*, 2009a, b). The chloroplastic FBPase (cFBP1; EC 3.1.3.11) is a key enzyme of the Calvin–Benson pathway and is involved in the regeneration of ribulose 1,5-bisphosphate (RuBP) and in the starch synthesis pathway.

cyFBP and cFBP1 display a similar tertiary structure, with the exception of an extra sequence of 20–30 amino acids in the regulatory domain of cFBP1 (called ‘loop 170’), which includes three cysteines, two of which can form disulphide bonds that can be reduced by plastidial thioredoxin *f* (TRX *f*) during light activation (Chiadmi *et al.*, 1999). The novel isoform cFBP2 lacks loop 170 in its sequence, is not redox regulated by TRX *f*, and its affinity for the substrate FBP is 6.6-fold lower than that of cFBP1 (Serrato *et al.*, 2009a, b). The activity of the cytosolic isoform is inhibited by an excess of substrate and shows allosteric inhibition by AMP and fructose-2,6-bisphosphate (F2,6BP). cyFBP and sucrose phosphate synthase (SPS) are considered major sites for controlling sucrose synthesis (MacRae and Lunn, 2006). Additionally, pyrophosphate:fructose-6-phosphate 1-phosphotransferase (PFP), which catalyses the reversible interconversion of F6P and F1,6BP, is also considered as an important regulatory point of primary carbon metabolism toward glycolysis or gluconeogenesis in the cytosol (Nielsen and Stitt, 2001).

Considerable effort has been made to investigate which steps control the biosynthesis and distribution of carbohydrates in plant cells. By using various transgenic approaches in different plant species, the roles of chloroplast and cytosolic FBPases have been analysed in this context (Koßman *et al.*, 1994; Sahrawy *et al.*, 2004). These results depended on the genetically manipulated plant species, the level of repression or overexpression of the gene selected (chloroplastic or cytosolic FBPase) (Sharkey *et al.*, 1992; Zrenner *et al.*, 1996; Strand *et al.*, 2000), and the tissue (leaf) or organ analysed (fruit or tuber) (Obiadalla-Ali *et al.*, 2004). Most of the data which have been reported concern the photosynthesis rate, starch and sucrose content, and general phenotypes. Nevertheless, these studies have not led to clear and consistent results on the specific role of FBPases. In general, the use of these transgenic strategies has given rise to some confusion on the function of FBPases in sucrose and starch levels in plants and their turnover, and the results to date remain imprecise, making it impossible to draw unambiguous conclusions.

To shed light on this confusing information, a comprehensive analysis of *Arabidopsis* cyFBP and cFBP1 loss-of-function mutants, as well as the corresponding double mutant has been performed for the first time. The main objective was to determine the contribution of each FBPase to photosynthesis, plant development, reactive oxygen species (ROS) metabolism, carbon partitioning, and metabolic profiles in leaves over a day/night period. Physiological, biochemical, and metabolic evidence is provided that cFBP1 activity is critical for normal plant development and important for a wide range of metabolic processes, while cyFBP appears essentially to affect starch levels.

## Materials and methods

### Plant material and growth conditions

*Arabidopsis thaliana* wild-type (ecotype Columbia) and mutant plants, *cyfbp*, line SALK_064456 (At1G43670) corresponding to the mutant line *fins1* (Cho and Yoo, 2011), and *cfbp1*, line GK-472G06-019879 (At3g54050) (Serrato *et al.*, 2009a), were grown in soil during 20 d in culture chambers under long-day conditions (16h light/8h darkness) at 22 °C during the light and 20 °C during darkness. The light intensity was set at 120 μmol m^–2^ s^–1^. The double mutant, called *cyfbp cfbp1*, was obtained by manual crossing of the single mutants *cyfbp* and *cfbp1*. The oligonucleotides used for the genotyping (Supplementary Table S1 available at *JXB* online) and the homozygous selections were: CYFBP F and R for *cyfbp*, and CFBP1 F and R for *cfbp1*, in conjunction with the oligonucleotides (LBSALK and GABI) hybridizing with the T-DNA sequence. Five plants were harvested at intervals of 5 d for 30 d, then the number of leaves per rosette was counted, and the fresh weight (FW) per plant and area were measured. For root length measurements, seedlings were grown in vertical plates. *Arabidopsis cfbp1* and *cyfbp* mutants were complemented with pGWB4-derived constructions expressing the green fluorescent protein (GFP) translationally fused proteins cFBP1:GFP and cyFBP:GFP under the control of 1kb of their respective promoters (Supplementary Fig. S1A).

### Gas exchange measurements and PSII photochemical efficiency

Photosynthetic gas exchange was measured using a portable LI-6400 infrared gas analyser (LI-COR Biosciences, Inc., Lincoln, NE, USA), which allows environmental conditions inside the chamber to be precisely controlled. The CO_2_ assimilation rate was determined in the upper leaf of the wild-type and mutant plants grown for 3 weeks by changing light intensities (light curve), with a range from 0 to 2000 μmol quanta m^–2^ s^–1^. To measure the CO_2_ response (CO_2_ curve), the CO_2_ concentration was changed with the range: 400 to 50 and 50 to 1500 μmol mol^–1^, and the irradiance was set at 1000 μmol quanta m^–2^ s^–1^. The photosynthetic parameters were calculated by using LI-6400 6.1 software. Photosyn Assistant, software developed by Dundee Scientific (Parsons and Ogston, 1999), was used to estimate the following parameters, dark respiration (*R*_d_), light compensation point (Г), and the maximum photosynthesis rate (*A*_max_), from the *A* to light (*A/Q*) curve as well as the maximum rate of Rubisco carboxylation (*V*_cmax_), maximum rate of electron transport (ETR) (*J*_max_), and TP use (TPU) from *A* to intercellular CO_2_ concentration (*A/C*_i_) curves, to help in the comparison between the mutants.

Parameters of chlorophyll fluorescence emission were measured at 22 ºC with a PAM 2000 chlorophyll fluorometer (Walz, Effeltrich, Germany). The maximum quantum yield of PSII (*F*_v_/*F*_m_) was calculated from the parameters using the following equation: *F*_v_/*F*_m_=(*F*_m_–*F*_o_)/*F*_m_, where *F*_o_ is the initial minimal fluorescence emitted from leaves dark adapted for 15min and *F*_m_ the maximal fluorescence elicited by saturating actinic light.

### Determination of photosynthetic pigments

After pigment extraction in 80% acetone, the content of chlorophyll *a* (Chl*a*) and *b* (Chl*b*), and carotenoids was spectrophotometrically quantified according to the method of Lichtenthaler and Wellburn (1983).

### Characterization of stomata

The shape and number of the stomata and epidermal cells were observed and measured from a similar leaf to that used for gas exchange determinations. Digital photographs of a 427-fold magnification were taken using a Zeiss variable pressure scanning electron microscope (LEO 1430VP) from six different fields per leaf of the adaxial and abaxial epidermis of three individual genotypes. Adobe Photoshop software was used for counting cell numbers and quantification of stomatal density.

### Oxidative metabolism assays

The H_2_O_2_ concentration in leaf extracts was measured by spectrofluorimetry using homovanillic acid (Ex=325nm and Em=425nm) and horseradish peroxidase as described elsewhere (Pazmiño *et al.*, 2011). The content of carbonyl groups was measured by derivatization with 2,4-dinitrophenylhydrazine, according to Romero-Puertas *et al.* (2002). Glycolate oxidase (GOX; EC 1.1.3.1) activity was assayed spectrophotometrically according to Kerr and Groves (1975). The activity of catalase (CAT; EC 1.11.1.6) was determined as described by Aebi (1984). Superoxide dismutase (SOD) isoenzymes were separated by native-PAGE on 10% acrylamide gels and were localized by a photochemical method (Beauchamp and Fridovich, 1971). Ascorbate peroxidase (APX; EC 1.11.1.11) activity was assayed as described by Jiménez *et al*. (1997). Lipid peroxidation was determined by the thiobarbituric acid-reactive substances method (Buege and Aust, 1972). Specific antibodies were used to determine 2-Cys peroxiredoxin (2-Cys Prx) and 2-Cys Prx-SO_2_H (oxidized form of 2-Cys Prx) proteins by western blotting (Iglesias-Baena *et al.*, 2010).

### Determination of sugars

Carbohydrates were extracted from frozen 20-day-old *Arabidopsis* leaf rosettes with 80% ethanol (v/v) at 80 ºC, followed by further washing with 50% ethanol at 80 ºC (Stitt *et al.*, 1978). After centrifugation, sucrose, glucose, and fructose were measured enzymatically in the extraction solution by determining the reduction of NADP at 340nm according to Sekin (1978). Starch was extracted with 50mM HEPES pH 7.6, 1% Triton X-100 buffer, and filtered through two layers of Miracloth (Millipore, MA, USA) and centrifuged. The pellet was resuspended in Percoll 90% (v/v), centrifuged and then the pellet was resuspended in ethanol and measured as glucose from the extract, following incubation with α-amylase and amyloglucosidase.

### RT–PCR analysis

Total RNA was extracted and reverse transcription–PCR (RT–PCR) was carried out as described by de Dios Barajas-Lopez *et al.* (2007). Primers used are listed in Supplementary Table S1 at *JXB* online.

### Light and electron microscopy

After sample processing (as described in de Dios Barajas-Lopez *et al.* (2007), semi-thin sections (1mm) of *Arabidopsis* leaves and roots were stained with toluidine blue for structure visualization in an OLYMPUS BX51 light microscope, and ultra-thin sections (70–90nm) were examined by high resolution transmission electron microscopy (TEM) (LIBRA 120-EDX**-**Carl Zeiss SMT).

### Protein extraction, western blotting, and FBPase and PFP enzymatic activities

The protein concentration of extracts was determined with the Bradford assay (1976). Western blotting and FBPase assays were performed according to Serrato *et al.* (2009a). The modified method of Kombrink (1984) was used to measure PFP activity.

### Measurement of hexose-phosphates, triose-phosphates, and 3-PGA

Leaf samples of the *Arabidopsis* wild type and mutants (six biological replicates) were snap-frozen in liquid nitrogen, ground to a fine powder using a liquid nitrogen-cooled Mixer Mill MM200 (Retsch; http://www.retsch.com), and extracted to measure hexose-phosphates (Glc6-P, Fru6-P, Glc1-P, and Fru1,6-BP), TPs [glyceraldehyde 3-phosphate (GAP) and dihydroxyacetone phosphate (DHAP)], and 3-phosphoglycerate (3-PGA) using enzymatic assays coupled to NAD(P)H fluorescence analysis, as previously described in Thormählen *et al*. (2013).

### GC-MS analysis of polar primary compounds

A 50mg aliquot of leaf samples prepared as above was extracted and the relative metabolite contents were determined by gas chromatography–mass spectrometry (GC-MS), as previously described in Thormahlen *et al*. (2013). For the visualization and analysis of networks with related experimental data, Vanted version 2.1.0 (IPK Gatersleben, Germany) was applied as a tool.

## Results

### T-DNA insertions knock out the expression of both FBPase isoforms

**Fig. 1.**
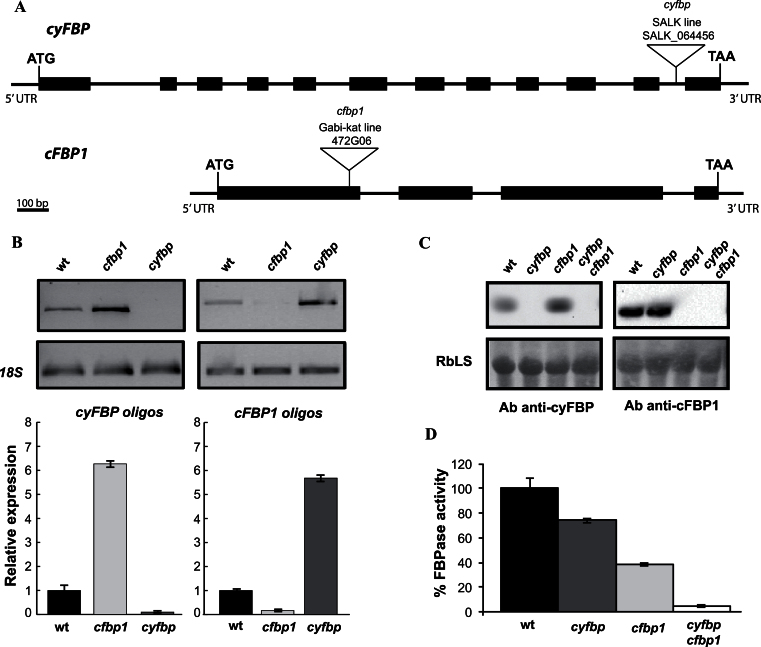
Analysis of mutant lines. (A) Genomic structure of *cyFBP* and *cFBP1*. Exons and introns are indicated as thick and thin black bars, respectively. Insertion sites of T-DNA in mutant lines *cyfbp* and *cfbp1* at intron 11 and exon 1, respectively, are indicated by triangles. (B) Expression profile of each FBPase using specific oligonucleotides in the *cyfbp* and *cfbp1* mutants and the wild type (wt); *18S* was used as the housekeeping gene. (C) Western blot analysis of crude leaf extracts of *cyfbp*, *cpfbp1*, and *cyfbp cfbp1* mutants and the wt. Proteins (25 μg) were separated by SDS–PAGEs, transferred to nitrocellulose filters, and immunolabelled with rabbit antiserum raised against cyFBPase and cFBPase1 antibodies (see the Materials and methods). Bands are ~40kDa, and RbcLS was used as loading control. (D) FBPase activity was determined in extracts of wt and mutants plants.

The T-DNA insertions are located in intron 11 and exon 1 (positions +1111 and +111 with respect to the start codon) for *cyfbp* and *cfbp1*, respectively (Fig. 1A). In Fig. 1B it can be seen that no *cyFBP* expression can be observed in the *cyfbp* mutant, corroborating previous results by Cho and Yoo (2011), and only a very faint *cFBP1* signal was observed in *cfbp1*. Interestingly, *cyFBP* transcript and protein increased in *cfbp1* whereas the amount of *cFBP1* mRNA was increased in the *cyfbp* mutant rosette (Fig. 1B, C). The double mutant *cyfbp cfbp1* was generated by crossing the respective single knockout mutants. In each case, the complete loss of the respective proteins (Fig. 1C) was confirmed by western blot analysis using specific antibodies (Serrato *et al.*, 2009a). The negligible *in vitro* FBPase activity in the double mutant validated the FBPase assay conditions, corroborating that the 80% of FBPase activity measured in *cyfbp* and the 40% of FBPase activity obtained in *cfbp1* were due to the cFBP1 and cyFBP activities, respectively (Fig. 1D). No compensation by PFP activity (using specific assay conditions of F1,6BP hydrolysis) was observed in any FBPase mutant, being similar in *cyfbp* and the wild type and surprisingly lower when cFBP1 was lacking (Supplementary Fig. S1C at *JXB* online).

### Changes in the phenotypes of cyfbp, cfbp1, and cyfbp cfbp1 mutants

**Fig. 2.**
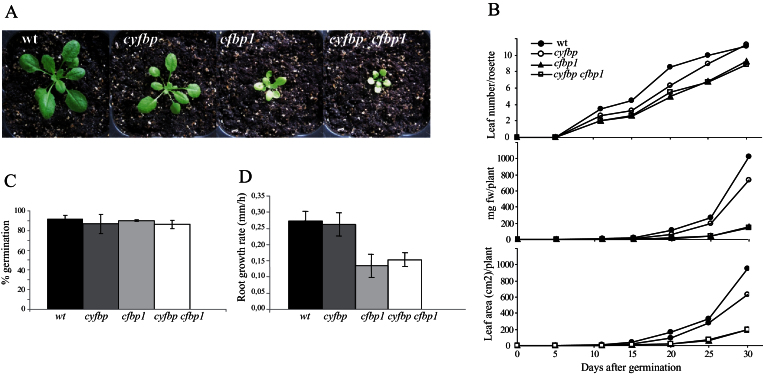
Growth of *cyfbp*, *cfbp1*, and *cyfbp cfbp1* mutants and the wild-type (wt) plants. Seeds of mutant and wt plants were sown in soil and cultured in a growth cabinet under 16h light/8h dark. Pictures were taken 21 d after sowing. (A) *cyfbp*, *cfbp1*, and *cyfbp cfbp1* mutants and the wt. (B) Leaf number per rosette; fresh weight (FW) in mg per plant, and leaf area in cm^2^ of mutants and the wt during the experimental time course plotted against the number of days after germination of seeds. (C) Rate of seed germination. (D) Root growth rate in the first 24h after germination.

The *cyfbp* mutant is slightly smaller than the wild-type plants (Fig. 2A) and no major difference is observed for this mutant. However, the absence of cFBP1 has a dramatic effect on plant development, and the rosettes of both *cfbp1* and the double mutant had fewer leaves, smaller size, and lower growth rates than the wild type (Fig. 2A, B). The fresh weight and leaf area decreased by 7-fold and 5-fold, in *cfbp1* and *cyfbp cfbp1* mutants, respectively, when compared with the wild type (Fig. 2B). Nevertheless, seed viability and germination were normal for all the mutants (Fig. 2C). Root growth analysis showed that *cfbp1* and *cyfbp cfbp1* roots were ~50% shorter and the root growth speed was 2-fold slower than that of wild-type and *cyfbp* roots (Fig. 2D).

**Fig. 3.**
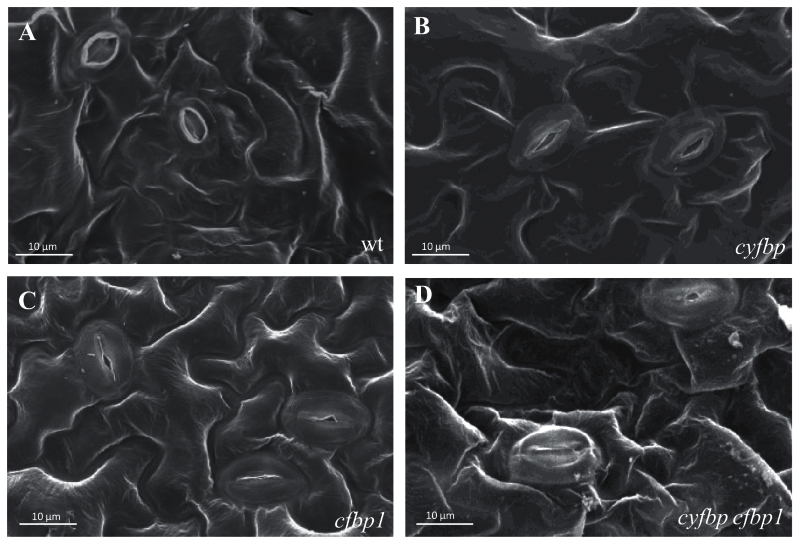
Scanning electron micrographs illustrating morphological differencesof the stomata of ‘adaxial’ leaf surfaces from *Arabidopsis* wild-type (wt) (A), *cyfbp* (B), *cfbp1* (C), and *cyfbp cfbp1* (D) mutants. Scale bars=10 μm.

Scanning electron microscopy analysis of the stomatal morphology of the abaxial side of *Arabidopsis* leaves showed a higher stomatal closure in *cfbp1* and double mutants compared with the full open stomata of wild-type plants under environmental conditions. As shown in Fig. 3B–D, and Supplementary Table S2 at *JXB* online, the stomatal density on the adaxial side of the *cyfbp*, *cfbp1* and *cyfbp cfbp1* mutants was 41, 23, and 29% lower, respectively, than that found on the adaxial surface of the wild-type leaves. However, the same mutants had 46%, 62%, and twice as many stomata per mm^2^ on the abaxial side than the wild type, respectively. With regard to leaf size, the stomatal index values of the rosette leaves of *cyfbp*, *cfbp1*, and *cyfbp cfbp1* were all lower than those of the wild type (Supplementary Table S2).

### Cell structure alterations of cyfbp, cfbp1, and cyfbp cfbp1 mutants

**Fig. 4.**
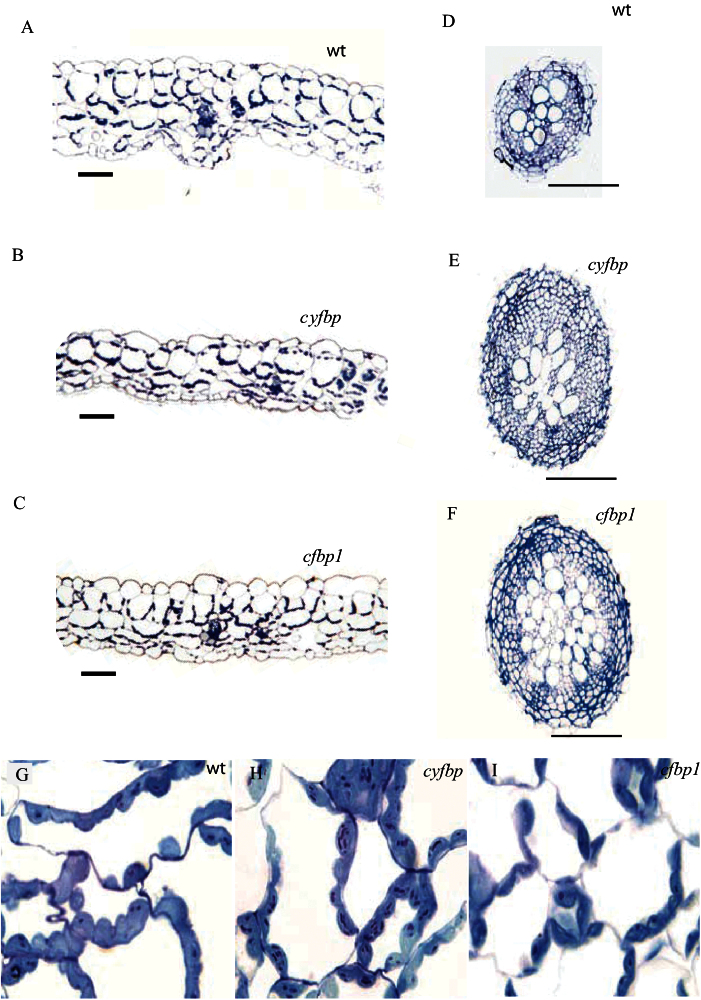
Light microscopy images of leaf cell types (A–C), the root vascular cylinders (D–F), and the cell structure (G–I) of the wild type (wt), and *cyfbp* and *cfbp1* mutants of *Arabidopsis* plants grown for 21 d under a 16h light/8h dark regime. Semi-thin cross-section of leaves and roots from the wt (A, D, G), *cyfbp* mutant (B, E, H), and *cfbp1* mutant (C, F, I) were stained with toluidine blue (which stains proteins). Scale bars=100 μm.

The structure of the non-flowering rosette leaf and root cross-sections analysed by light microscopy showed different cell types in leaves, epidermis, mesophyll (palisade and spongy), xylem, phloem, and stomata (Fig. 4A–C). The cell structure of the *cyfbp* mutant is similar to that of the control plant, but chloroplasts contained more starch granules when examined at a higher magnification (Fig. 4G–I). The *cfbp1* mutant had a higher number of intercellular spaces, and few chloroplasts with fewer starch granules (only one in some cases) (Fig. 4I). Some of the chloroplasts displayed a centrifugal position only on opposite side to the light source (Fig. 4C). *cyfbp cfbp1* showed similar cell structure to its *cfbp1* parent (data not shown).

The *cfbp1* mutation resulted in a greater number of cell layers in the root vascular cylinder, and there were twice as many vascular tissue cells per layer, in comparison with the wild-type root (Fig. 4D, F). However, no disorganization was detected and the shape and size of cells were normal. The roots of the *cyfbp* mutant had a slightly higher number of cells in the vascular cylinder than the control (Fig. 4D, E).

**Fig. 5.**
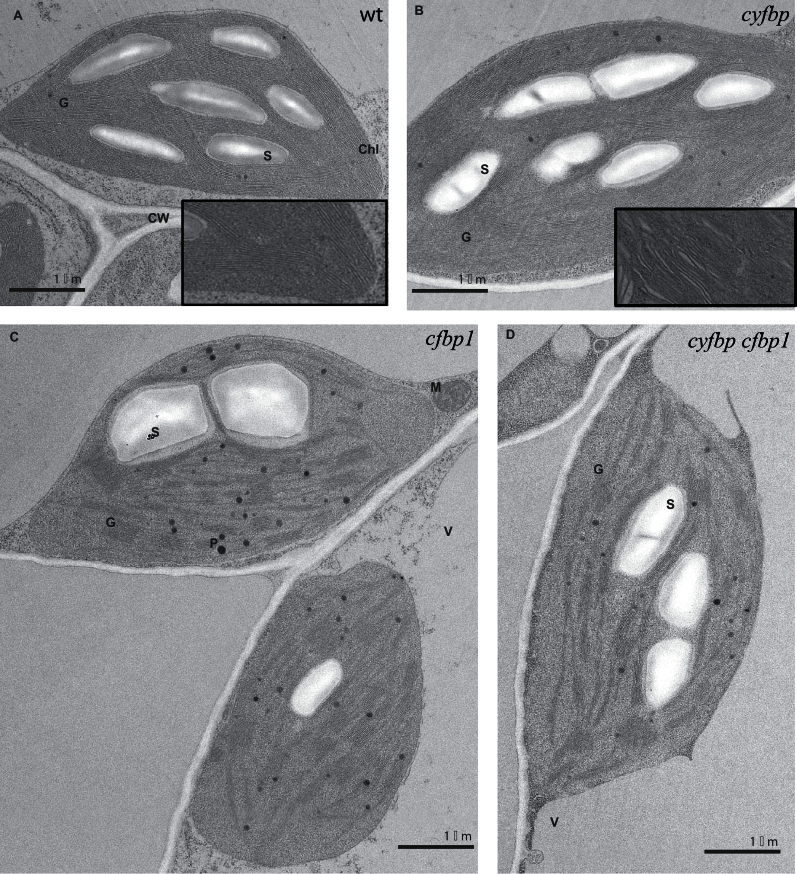
Transmission electron microscopy analysis of leaf sections from wild- type (wt) (A), *cyfbp1* (B), *cfbp1* (C), and *cyfbp cfbp1* (D) plants. Leaves were collected at 4h in a 16h light/8h dark photoperiod, fixed, embedded, and sectioned as described in the Materials and methods. G, grana; S, starch; V, vacuole; P, plastoglobule; Chl, chloroplast; CW, cell wall; M, mitochondrion.

The observations by TEM showed disturbances in the cell structure of the *cfbp1* and double mutant, characterized by a decreased number of thylakoids and grana lamellae but without disrupting the chloroplast ultrastructure (Fig. 5). A higher number of plastoglobuli was detected in *cfbp1* and *cyfbp cfbp1* chloroplast than in the wild type (Fig. 5C, D). A lower starch content was observed in *cfbp1* and the double mutant than in *cyfbp* and the wild type (Fig. 5A, B).

### Pigment content decreases drastically in cfbp1 and cyfbp cfbp1

**Fig. 6.**
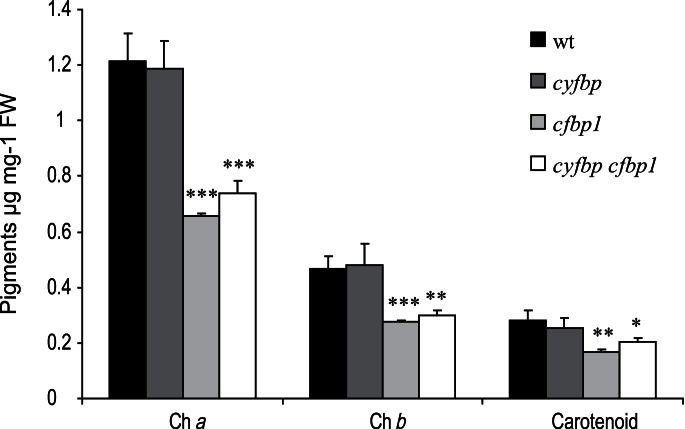
Pigment content of chlorophyll *a* and *b* and carotenoids in wild-type (wt) and *cyfbp*, *cfbp1*, and *cyfbp cfbp1* plants. Values are means ±SE of measurements on at least 5–7 leaves of three different plants. Error bars show the standard error of the squared mean. Significant differences between means within a time point are indicated with asterisks (*P<0.05, **P<0.01, ***P<0.001).

The Chl*a*, Chl*b*, and carotenoid contents were considerably reduced (~40–50%) in the *cfbp1* and *cyfbp cfbp1* mutants compared with the wild type, while the *cyfbp* mutant displayed values similar to those of the wild type (Fig. 6).

### Effect of FBPase removal on CO_2_ assimilation and PSII photochemical efficiency

**Fig. 7.**
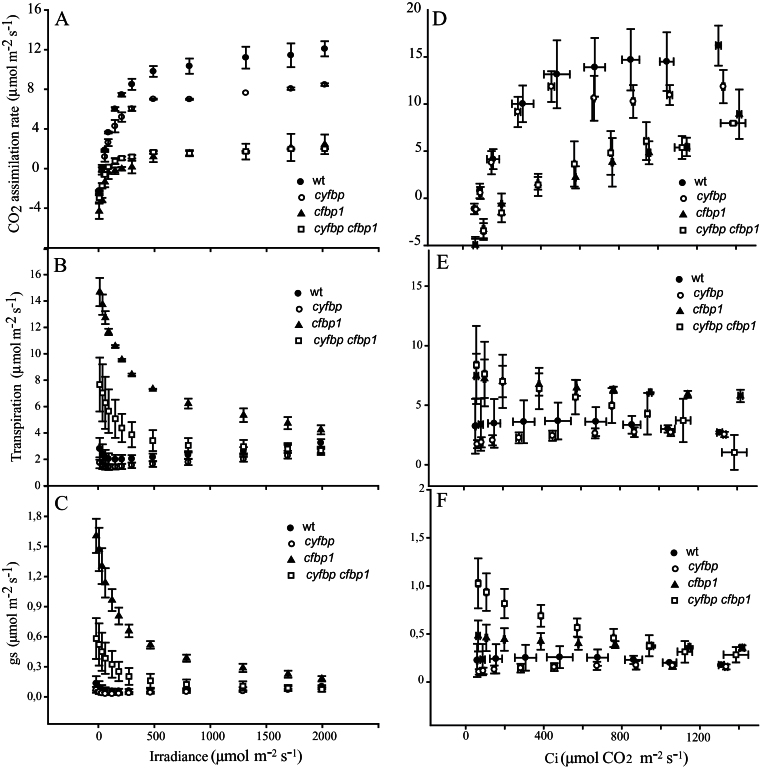
Photosynthetic capacity of the wild type (wt) and *cyfbp*, c*pfbp1*, and *cyfbp cpfbp1* mutants. Plants were grown in a controlled growth cabinet under 100 μmol m^–2^ s^–1^ light regimes for 20 d. Photosynthetic carbon fixation rates were determined in the newest fully expanded leaf, as a function of increasing irradiance (A) at saturating CO_2_ (400 μmol mol^–1^; *A*/*Q* response curve) and as a function of increasing CO_2_ concentration (D) at saturating light levels (1000 μmol m^–2^ s^–1^; *A*/*C*_i_ response curve). Transpiration (*E*) and stomatal conductance (*g*_s_) were determined in the same leaves (B, E and C, F). Values represent the mean of eight plants ±SE.

With an open gas exchange system, CO_2_ assimilation rates (*A*) were determined on attached leaves of plants grown under 120 μmol m^–2^ s^–1^ and ambient CO_2_. The light–response curves (*A*/*Q*) at ambient CO_2_ are shown in Fig. 7A. Under these conditions, the photosynthesis rate of the wild type had a maximum of 13.5 μmol m^–2^ s^–1^ at 2000 μmol m^–2^ s^–1^. At light intensities <250 μmol m^–2^ s^–1^, the *cyfbp* mutant assimilation rate was similar to that of wild-type plants, while at higher intensities the assimilation rate was ~33% lower. At light intensities between 100 μmol m^–2^ s^–1^ and 500 μmol m^–2^ s^–1^ (standard growth conditions), the *A* of the *cfbp1* and *cyfbp cfbp1* mutants showed superimposed curves with values near 1, indicating an impaired CO_2_ assimilation capacity and a poor photosynthesis/respiration ratio. At higher intensities, both *cfbp*1-containing mutants had lower CO_2_ fixation (~6-fold) than did the wild type, reaching a maximum of 3.4 μmol m^–2^ s^–1^ (Fig. 7A). Transpiration (*E*) and stomatal conductance (*g*_s_) values were higher at lower irradiance in *cfbp1* and *cyfbp cpfbp1* leaves in comparison with wild-type plants (Fig. 7B, C). However, when the light intensity was increased, *E* and *g*_s_ converged to reach wild-type and *cyfbp* mutant values at 2000 μmol m^–2^ s^–1^ (Fig. 7B, C).

The response of net photosynthesis to increasing internal leaf CO_2_ concentration (*C*_i_) at 1000 μmol m^–2^ s^–1^ (*A*/*C*_i_ curve) exhibited a similar behaviour (Fig. 7D). The photosynthesis rate of wild-type plants increased to a maximum of 16.2 at 1300 ppm of CO_2_. At the same concentration of CO_2_, the *cyfbp* mutant decreased by 25%, whereas *cfbp1* and *cyfbp cfbp1 Arabidopsis* plants had a 2-fold lower photosynthetic rate. This suggests that cFBP1 deficiency exerts a stronger effect on CO_2_ fixation than cyFBP deficiency. Transpiration values of *cfbp1* mutant were higher than those of the other mutant lines and control plants at all CO_2_ concentrations tested (Fig. 7E). Curiously, transpiration and conductance in the *cyfbp cfbp1* mutant (Fig. 7E, F) displayed high values at lower CO_2_ concentrations but these declined slowly at higher concentrations, reaching levels similar to those of the wild-type plants. No significant differences were detected for the *E* and *g*_s_ of the wild type and *cyfbp* mutant in relation to the intercellular CO_2_ concentration.

The Photosyn Assistant program, version 1.1.2, which is based on the von Caemmerer and Farquhar equations (von Caemmerer and Farquhar, 1981) (Table 1), was used to help in the interpretation, comparison, and modelling of photosynthesis of plants grown under different environmental conditions. Based on *A*/*Q* curves, it was found that respiration rates (*R*_d_) increased by 1.6- and 1.3-fold in *cfbp1* and the double mutant, respectively, compared with wild-type plants, but the differences were not significant (Table 1). The light compensation point exhibited higher values in *cfbp1* and the double mutant, but only *cfbp1* data differed significantly. The *A*_max_ of *cfbp1* and *cyfbp cfbp1* was 2- and 3-fold lower, respectively, than that of the wild type and *cyfbp*, as indicated in the *A*/*Q* curves (Fig. 7).

**Table 1.** Photosynthetic parameters of wild-type, cyfbp, cfbp1, and cyfbp cfbp1 mutant plants Values were obtained from the *A*/*Q* and *A*/*C*_i_ curves using the Photosyn Assistant software as described in the Materials and methods.

	*A*_max_	Γ	*R*_d_	*V*_cmax_	*J*_max_	TPU	*F*_v_/*F*_m_
Wild type	9.5±0.5	14.6±1.5	–1.1±0.3	30.6±4.9	154±33	11.1±1.4	0.83±0.01
*cyfbp*	9.3±0.5	16.7±5.4	–1.25±0.0	31.1±4.2	143±25	10.1±1.4	0.83±0.01*
*cfbp1*	4.2±0.4**	67.3±23*	–1.81±0.7	19.4±2.1*	88±12*	8.4±1.2*	0.77±0.01***
*cyfbp cfbp1*	3.2±0.7 **	50.4±31	–1.41±0.5	20.1±4.0	92±23*	8.4±1.2*	0.77±0.03***

Values are the mean ±SE of 5–10 independent determinations.

*F*_v_/*F*_m_ was determined in 10 leaves from different plants. Values are the mean ±SD.

Asterisks indicate that mean values are significantly different between wild-type and FBPase mutant plants (*P<0.05; **P<0.01; ***P<0.001).

The response of net CO_2_ uptake to increasing intercellular CO_2_ (*C*_i_), the *A*/*C*_i_ curve, showed clear differences between *cfbp1* and the double mutant compared with wild-type plants. A lack of plastidial FBPase and of both FBPases led to a decrease of 37% and 34% in *V*_cmax_, 42% and 40% in *J*_max_, and 25% and 25% in TPU, respectively, suggesting damage to the CO_2_ assimilation process (Table 2). In contrast, the values of both *J*_max_ and *V*_cmax_ of plants lacking only cyFBP were similar to control values.

**Table 2.** *Sucrose/starch ratio in leaves of the* Arabidopsis *wild type, and cyfbp, cfbp1, and cyfbp cfbp1 mutants*

Hours	Wild type	*cyfbp*	*cfbp1*	*cyfbp cfbp1*
0	0.87	0.66	5.15	1.91
4	1.11	0.48	3.18	1.05
8	1.39	0.29	1.89	0.20
12	0.72	0.67	2.85	1.14
16	1.02	0.73	1.42	0.96
20	1.16	0.91	3.00	3.02

The chlorophyll fluorescence analysis of PSII (*F*_v_/*F*_m_) showed a significant decrease of the photochemical performance for the *cfbp1* and *cyfbp cfbp1* mutants (Table 1), indicating a lower quantum efficiency of linear electron transport through PSII in these two mutants, in agreement with the above *J*_max_ data.

### Oxidative metabolism in the mutants

**Fig. 8.**
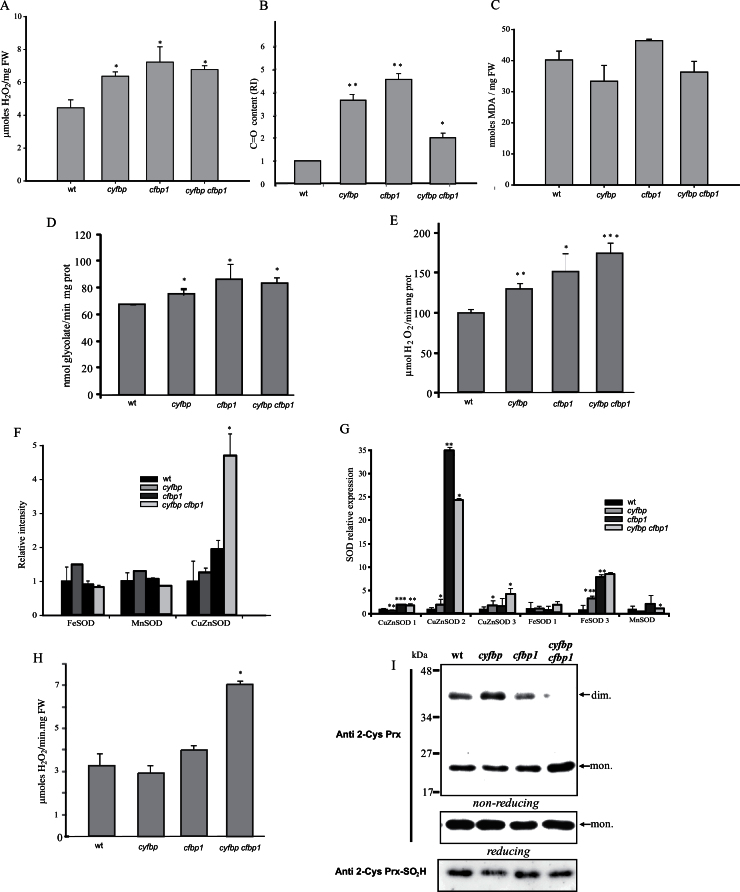
Reactive oxygen species metabolism in the *Arabidopsis* wild type (wt) and *cyfbp*, *cfbp1*, and *cyfbp cfbp1* mutants. (A) Determination of H_2_O_2_ by fluorometry. (B) Protein oxidation measured as carbonyl group content. (C) Lipid peroxidation measured as malondialdehyde (MDA) content. (D) Glycolate oxidase (GOX) activity. (E) Catalase (CAT) activity. (F) Relative intensity of MnSOD, FeSOD, and CuZnSOD activities quantified by the Bio-Rad software Quantity one. (G) Analysis of mRNA SOD expression by qRT–PCR. (H) Ascorbate peroxidase (APX) activity. (I) Western blot analysis using anti-2-Cys Prx and anti 2-Cys Prx-SO_2_H antibodies. Each bar represents the mean ± SE of three independent experiments. Differences between mutant plants and the wt were significant at *P<0.05, **P<0.01, and ***P<0.001.

FBPase removal affects ROS metabolism in *Arabidopsis* mutants, and resulted in an increase in H_2_O_2_ accumulation by 42, 60, and 51%, in *cyfbp*, *cfbp1*, and double mutants, respectively (Fig. 8A). The level of protein carbonyl groups also increased by 4-, 9-, and 2-fold in *cyfbp*, *cfbp1*, and *cyfbp cfbp1*, respectively (Fig. 8B). However, lipid peroxidation did not change significantly in any of the mutants compared with the wild type (Fig. 8C). In order to establish possible sources of H_2_O_2_, two enzymes were studied, GOX, an enzyme from the photorespiratory pathway in peroxisomes, and SOD, which removes O_2_·^−^ and at the same time produces H_2_O_2_. GOX activity increased in the three mutants, with the level in *cfbp1* being the highest (1.3-fold; Fig. 8D). CAT, a peroxisomal protein involved in the detoxification of H_2_O_2_, showed a significant increase in all the lines, but mainly in *cfbp1* (1.5-fold) and the double mutant (1.8-fold; Fig. 8E). However, the increase in CAT was insufficient to avoid protein oxidative damage, although in the double mutant this increase is lower. The analysis of SOD isoform activity showed a low increase in FeSOD and MnSOD in *cyfbp*, and a strong induction of CuZnSOD in *cfbp1* and *cyfbp cfbp1* (Fig. 8F). Expression analysis revealed a significant induction of the plastid isoforms CuZnSOD2 and FeSOD3 in all mutants, the highest changes being in the *cfbp1* lines (Fig. 8G). APX, also involved in H_2_O_2_ removal and present in all chloroplasts and the cytosol, is induced in *cyfbp cfbp1* (Fig 8H). Western blotting analysis using anti-2-Cys Prx showed a lower amount of dimer forms (Fig. 8I) and similar expression in reducing conditions of all the mutants compared with the control plant (Fig. 8I). In addition, 2-Cys Prx-SO_2_H was lower in all the mutants, as observed in western blotting (Fig. 8I). No differences in the non-enzymatic antioxidants ascorbate and dehydroascorbate were found after 8h light, although the ASC/DHA ratio increased in *cfbp1* and the double mutant and decreased in *cyfbp* (Supplementary Tables S3, S4 at *JXB* online).

### Day/night cycle of carbohydrate accumulation in cyfbp, cfbp1, and cyfbp cfbp1

**Fig. 9.**
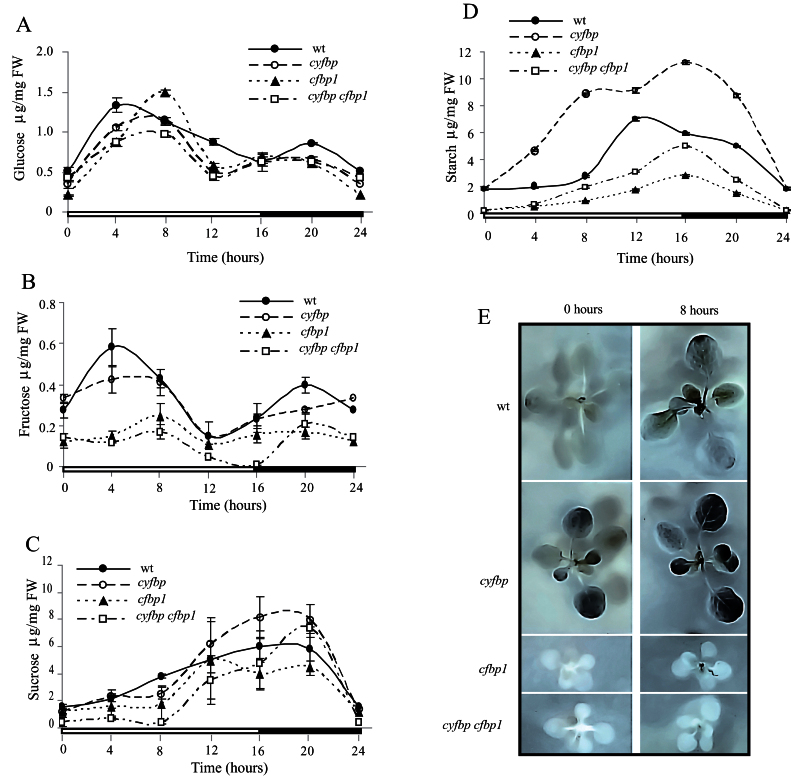
Changes in the intracellular content of glucose (A), fructose (B), sucrose (C), and starch (D) in wild-type (wt), *cyfbp*, *cfbp1*, and *cyfbp cfbp1* mutant plants. The rosette leaves were collected at 0, 4, 8, 12, 16, and 20h (4h dark) of the photoperiod. The results are the mean ±SE from three individual *Arabidopsis* rosettes of three different experiments. (E) Plantlets of wt and mutants plants were harvested after 8h illumination and at the end of the night, and stained in Lugol solution.

The level of soluble sugars, such as glucose, fructose, and sucrose, in wild-type and mutant plants was analysed every 4h over a 24h period (Fig. 9). Glucose accumulated during the central light period in all the lines and declined as darkness approached, but the concentrations were slightly lower than in the wild-type line (Fig. 9A). Compared with wild-type plants, all FBPase mutants showed a 4h delayed glucose accumulation peak at 8h and a dramatic drop 4h before the end of the day (the wild type displaying a constant and negative slope from 4h to 16h). *cyfbp* showed similar fructose amounts to the wild type, except at the points corresponding to the wild-type peak-like shape at 4h and 20h (Fig. 9B), whilst this amount decreased drastically in the *cfbp1* background. The wild-type and *cyfbp* plants accumulated fructose at the beginning of the light period; this diminished after 8h illumination, while the *cfbp1* fructose concentration was almost constant over the photoperiod (~0.2 μg mg^–1^ FW). The fructose level in the *cyfbp cfbp1* leaves fell sharply (fructose depletion) at 16h, followed by a sharp rise during the period coinciding with starch degradation. In FBPase mutant lines, the inflexion point in sucrose accumulation occurs at 8h of the light period (slightly before that in the wild type), reaching maximum accumulation during the first half of the night period, and decreasing rapidly at the end of this period, with a similar profile in all plant lines (Fig. 9C). *cfbp1* had a lower sucrose content over the photoperiod, and although *cyfbp cfbp1* showed a similar profile to *cfbp1* at the beginning of the light period, there was a gradual recovery ending with an accumulation peak close to that of the sucrose concentration in the wild type at night (20h).

It was noteworthy that after 8h illumination, *cyfbp* mutant plants displayed a starch content that was ~4-fold higher than in the wild type (Fig. 9D). In contrast, the starch content was ~3-fold lower in *cfbp1* compared with that found in control plants. The amount of starch detected for the double mutant plants was double that of the *cfbp1* mutant, but lower than in the wild type and *cyfbp*, revealing that the low quantity of starch accumulated is not exclusively due to a limitation in the carbon fixation capacity of *cfbp1*. Staining plantlets with Lugol solution confirmed the higher starch accumulation in the *cyfbp* mutant (Fig. 9E). The foliar sucrose/starch ratio, an indicator of photoassimilate allocation (Table 2), was positive toward the starch content (<1) for the *cyfbp* line throughout the photoperiod, indicating an increased starch content in the chloroplasts when cyFBP is lacking. Conversely, this ratio was balanced towards sucrose synthesis in *cfbp1* (>1), while in *cyfbp cfbp1* the ratios were lower and even less than 1.

### Effect of the loss of cyFBP, cFBP1, and of both FBPases on leaf metabolite levels

Supplementary Tables S3 and S4 at *JXB* online show the changes in leaf metabolite levels at the end of the night and after 8h of illumination (middle of the day). The lack of cyFBP activity induced a slight decrease in sugars during the night period, with the exception of maltose and trehalose, while most sugars increased in the light period, with the rise in isomaltose content being statistically significant. The *cfbp1* and *cyfbp cfbp1* mutant plants in the light and at the end of the night showed between 50% and 90% decreases in comparison with the wild-type in the levels of most of the sugars analysed, such as sucrose, glucose, fructose, isomaltose, and trehalose, suggesting a strong impairment of the Calvin–Benson cycle.

As expected, the lack of cytosolic or chloropastic FBPases during the day led to an accumulation of F1,6BP content, which was ~4-, 17-, and 60-fold higher in *cfbp1*, *cyfbp*, and *cyfbp cfbp1*, respectively (Fig. 11; Supplementary Table S4 at *JXB* online). A significant increase in TPs (DHAP and GAP) was observed, mainly in the double mutant, and in 3-PGA, the first carbon assimilates synthesized after CO_2_ fixation and reduction (Supplementary Table S4 at *JXB* online). At the end of the night, the hexose-phosphate and DHAP pools declined sharply in all the mutants. During the light period, the level of 3-PGA increased in all mutants, principally in the *cyfbp* mutant, and a marked decrease ocurred in *cfbp1* and *cyfbp cfbp1* in the night (Supplementary Table S3).

Interestingly, the lack of cFBP1 led to marked changes in the levels of organic acids. As revealed in Supplementary Tables S3 and S4 at *JXB* online, the organic acid level decreased after 8h light and more intensely during the night period, especially in the *cfbp1* and *cyfbp cfbp1* mutants. It is worth mentioning the low content detected of glycerate, malate, fumarate, gluconate, succinate, and threonate in the metabolite group, indicating possible effects on the tricarboxylic acid (TCA) cycle in the mitochondria. During illumination, the *cyfbp* mutant displayed an increase in gluconate and gulonate content.

Lack of cFBP1 also led to changes in the levels of amino acids. Threonine increased by 16- and 8-fold during the night and by 9- and 5-fold at midday in the *cfbp1* and *cyfbp cfbp1* lines, respectively, compared with the wild-type leaf. After 8h of light, *cfbp1* registered an increase in glycine and proline. Meanwhile, the amounts of serine and leucine in *cfbp1* and double mutant plants reached half the values found in the wild type. The changes in the levels of glycine, serine, and glycerate suggest that photorespiration is strongly affected. In contrast, only the level of aspartate increased (3-fold) during the night in the *cyfbp* line.

During the light period, the group of sugar alcohol metabolites increased in the *cyfbp* line and generally decreased in *cfbp1* and *cyfbp cfbp1* (Supplementary Table S4 at *JXB* online). However, the erythritol, glycerol, myo-inositol, and maltitol contents were significantly different in the *cfbp1* and double mutant lines. The higher content of ascorbate by 3- and 5-fold in *cfbp1* and *cyfbp cfbp1* mutant lines, respectively, is interesting when compared with the wild type during the night. The *cyfbp* mutant also displayed an increase in dehydroascorbate, a product of the ascorbic acid pathway. This suggests changes in the redox status and the possible activation of a detoxifying mechanism.

**Fig. 10.**
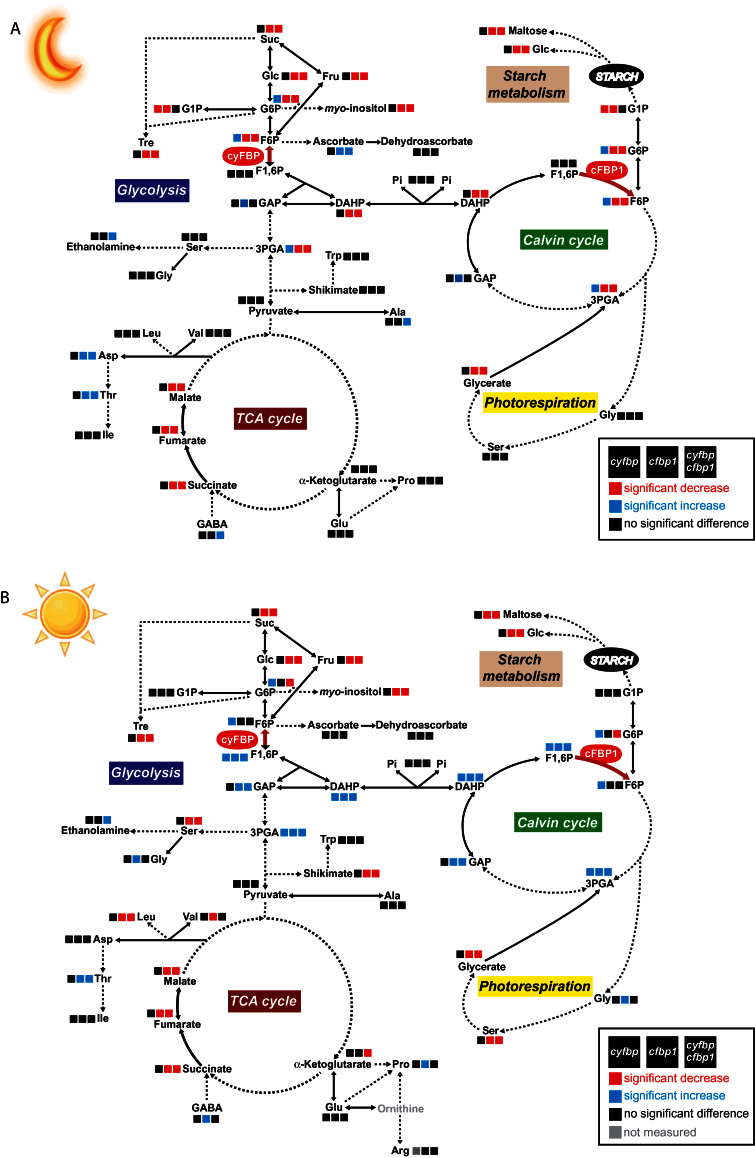
Summary of metabolite profiling of leaves at the end of the night (A) and after 8h of light (B) as analysed by gas chromatography coupled with mass spectroscopy (GC-MS) and enzymatic assays coupled to fluorescence spectroscopy. Ratios are given between *cyfbp*, *cfbp1*, *cyfbp cfbp1*, and the wild-type in colour coding: red, significantly lower than the wt; blue, significantly higher than the wt (Student’s t-test, *P*<0.05, *n*=6). For the data set of metabolite profiling see Supplementary Tables S3 and S4 at *JXB* online.

The Vanted diagrams provide an overview map of the clear metabolic changes in *cyfbp*, *cfbp1*, and *cyfbp cfbp1* at the end of the night (Fig. 10A) and during the light period (Fig. 10B). These diagrams reveal that the lack of different FBPase isoforms disturbs various central metabolic processes, affecting the plant physiology and the development, as shown above.

**Fig. 11.**
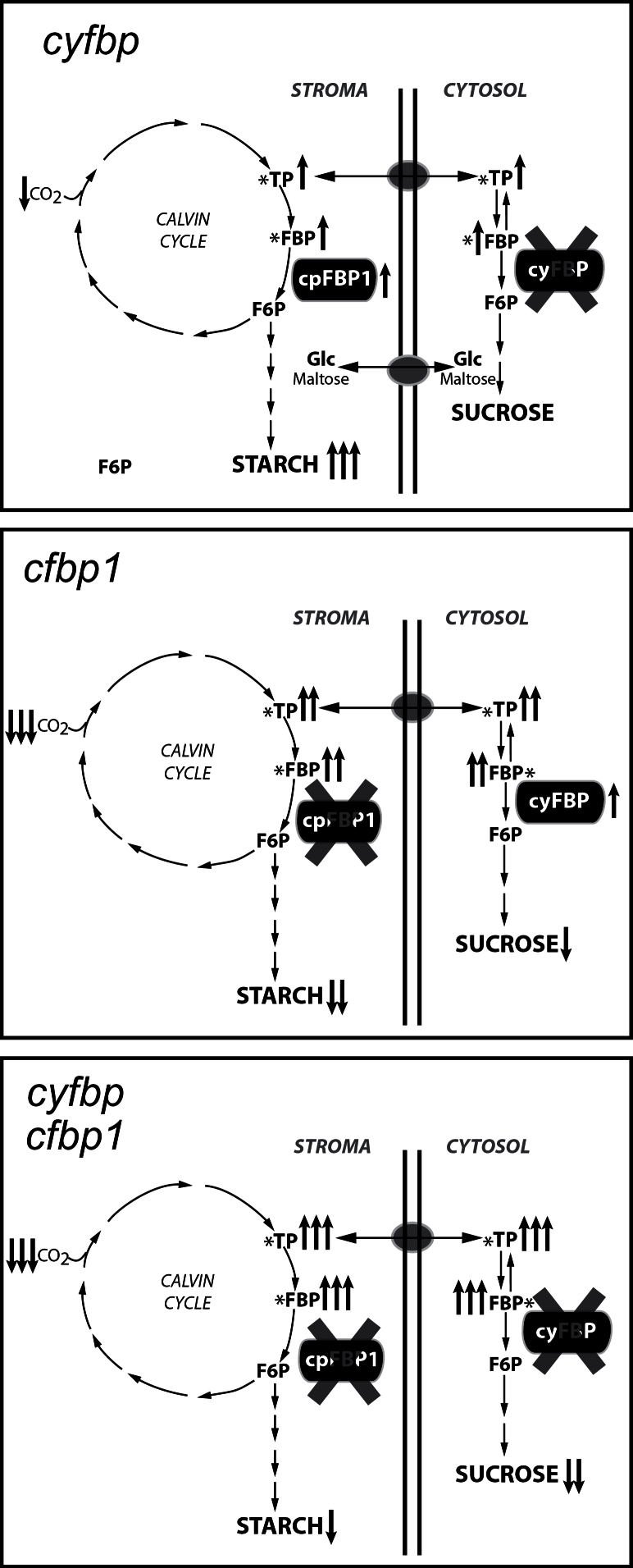
Simplified scheme of the major changes (in metabolism, photosynthesis, and gene regulation) occurring in FBPase mutants and their impact on accumulation of end-products (sucrose and starch). TP, trioses phosphate; F1,6BP, fructose-1,6-phosphate; F6P, fructose-6-phosphate.

## Discussion

The existence of different FBPases in plants makes it difficult to predict the precise role or the specific metabolic contribution of each of the isoforms. In previous studies using the antisense strategy in *A. thaliana* and other plant species (potato, tomato, or rice), several authors reported that FBPases play an important role during the regulation of primary photosynthetic metabolism and carbohydrate synthesis in plants (Koßman *et al.*, 1994; Obiadalla-Ali *et al.*, 2004; Lee *et al.*, 2008). However, most of the results were dissimilar and confusing, possibly due to specific plant metabolic adaptations in response to particular life cycles or environmental conditions.

The aim of this work is to understand the inter-relationship between the two main gluconeogenic pathways and the overall contribution of the FBPase isoforms cyFBP and cFBP1 through the study of three *Arabidopsis* mutants: *cyfbp* (affecting sucrose synthesis), *cfbp1* (affecting the Calvin–Benson cycle/starch synthesis), and *cyfbp cfbp1*. While Cho and Yo (2011) reported the role of *fins1* (*cyfbp*) in fructose signalling, in this study a comprehensive physiological and metabolic characterization of this mutant was carried out under normal growth conditions, together with analysis of the *cfbp1* mutant and the line obtained by combining both FBPase mutations. The null *cyfbp* mutant showed a normal phenotype and plant growth was only slightly affected, indicating that, in *Arabidopsis*, sucrose synthesis may be possible with hexoses or hexose-phosphates exported from the chloroplasts (Fettke *et al.*, 2011), probably due to an enhanced starch turnover. Several results obtained with this mutant support this hypothesis: there is (i) no compensation of FBPase activity by the cytosolic PFP (Supplementary Fig. S1C at *JXB* online); (ii) a starch overaccumulation (Fig. 9D, E) and a higher content of starch degradation products (Supplementary Tables S3, S4); and (iii) an up-regulated expression of the maltose transporter (*MEX1*), the plastidic glucose translocator (*pGlcT*), and the glucose 6-phosphate/phosphate translocator 1 (*GPT1*) and 2 (*GPT2*) (Supplementary Fig. S2A) (Cho *et al.*, 2011). In a similar way, the lack of the TP translocator in the *Arabidopsis tpt-2* mutant (blocking TP export into the cytosol) induces a higher starch accumulation compared with wild-type plants, but maintains a similar sucrose content. Interestingly, despite showing a non-altered sucrose level, cyFBP is down-regulated in *tpt-2* (Supplementary Fig. S2B) (Cho *et al.*, 2011). All these results suggest that *A. thaliana* could circumvent the cytosolic gluconeogenic pathway by accumulating and mobilizing more starch to export hexose/hexose-phosphates from the chloroplast to the cytosol with only a slight loss in photosynthetic efficiency. In contrast to cyFBP, the absence of cFBP1 leads to a dramatic phenotype, suggesting the impairment of many physiological processes, mainly photosynthesis and CO_2_ fixation, as has been shown in this work; with cyFBP up-regulation not being sufficient to compensate the cFBP1 loss (Fig. 1B, C). The original hypothesis of this study presumed that an additive/synergic negative effect of the two mutations on cell gluconeogenesis might have led to a lethal condition. However, surprisingly, *cyfbp cfbp1* is viable, displaying a *cfbp1* phenotype (Fig. 2). Expression experiments revealed only a slightly higher transcript accumulation of the plastidial isoform *cFBP2* in *cyfbp cfbp1* (Fig. S1B at *JXB* online). However, observing the negligible FBPase activity in this mutant, the contribution of cFBP2 and PFP seems to be very limited (Supplementary Fig. S1C). Moreover, the lack of *cFBP2* induction in *cfbp1* makes the substitution of the cFBP1 function in the chloroplast unlikely (Supplementary Fig. S1B).

The chlorotic aspect of *cfbp1* and *cyfbp cfbp1* leaves reflects the fact that the mutation directly determines the photosynthetic potential and primary production in these mutants. Therefore, the chlorophyll fluorescence results indicating that the lack of *cFBP1* affected PSII and the photosynthetic electron transport rates are in line with the findings of a previous study in which the authors describe an *Arabidopsis* mutant with a loss-of-function allelic variant of *cFBP1* (*hcef1*, from *high cyclic electron flow 1*), which constitutively induces cyclic electron flow (CEFI) to balance the chloroplast energy budget (Livingston *et al.*, 2010). The decline of PSII efficiency and the rate of photosynthetic electron transport (*J*_max_, based on NADPH requirement) for *cfbp1* and *cyfbp cfbp1* indicates that CO_2_ assimilation was also limited by the rate of electron transport and RuBP regeneration. Furthermore, removal of chloroplastic FBPase activity led to a decrease in *V*_cmax_ and TPU, and, consequently, as corroborated by the metabolite analysis, an increasing accumulation of TPs in all mutant lines (especially in *cfbp1* and *cyfbp cfbp1*). It seems that the drastic changes in the organic acids malate, fumarate, and succinate may lead to the stomatal failure of the *cfbp1* mutant epidermis at midday (Fig. 3), resulting in a limited CO_2_ gas exchange (Driscoll *et al.*, 2006; Araujo *et al.*, 2011; Zheng *et al.*, 2013). No relevant difference in the rate of photosynthesis was detected in the case of the *cyfbp* line.

Surprisingly, a greater number of cell layers in the root vascular cylinder of *cfbp1* root tissues in comparison with the wild type was observed, suggesting that root ontogenic factors are involved in order to counteract the metabolism imbalance, highlighting the co-ordination existing between green and non-green organs. It would be interesting to know what the physiological significance of this root remodelling is and the nature of the factors involved (i.e. hormones and/or transcription factors). To counteract the photosynthesis and carbohydrate metabolism deficiencies, *cfbp1* root tissues might activate different metabolic pathways. Soto *et al.* (unpublished results) identified double the number of genes expressed in this tissue compared with the wild-type plant in a transcriptomic analysis of both FBPase knockout mutants.

In addition, photosynthetic light reactions providing the NADPH and ATP necessary for CO_2_ fixation and carbohydrate synthesis inevitably are linked to the production of harmful ROS (Mittler, 2002). Thus, alterations observed in PSII and the Calvin–Benson cycle in *cfbp1* and *cyfbp cfbp1* mutants led to ROS production. Under these circumstances oxygen can be the final acceptor of electrons from the photosynthetic electron transport chain giving rise to O_2_·^–^ accumulation in chloroplasts. This situation was confirmed by the strong induction of CuZnSOD2 and FeSOD3 (Fig. 8G, H) (Kliebenstein *et al.*, 1998). Another source of ROS is the GOX associated with photorespiration in peroxisomes which was induced in all mutant lines. The accumulation of H_2_O_2_ in these organelles induced CAT (Fig. 8E), although this did not prevent oxidative damage. It appears that H_2_O_2_ is mainly produced in the chloroplast and peroxisomes, although other sources of ROS such as NADPH oxidases or mitochondrial electron transport cannot be ruled out. Despite the fact that APX activity is induced in the double mutant, western blotting analysis (Fig. 8I) shows that the plastidial antioxidants 2-Cys Prxs are not overoxidized in the FBPase mutants, indicating that the chloroplasts may not be under a high oxidative stress (Iglesias-Baena *et al.*, 2010). In addition, lack of both FBPases, mainly cFBP1, provokes changes in ROS metabolism and triggers an adjustment of the ASC/DHA ratio as a detoxifying mechanism. The protein targets of carbonyl production include those involved in photosynthetic CO_2_ assimilation and photorespiratory carbon oxidation (Rubisco large and small subunit and Rubisco activase); light-induced water oxidation at PSII (OEC3); and light harvesting and energy transfer at the photochemical reaction centre (Chl *a*/*b*-binding protein) (Johansson *et al.*, 2004).

The comprehensive GC-TOF MS and fluorescence spectroscopy metabolite analysis provided an overview of visible metabolic alteration in the FBPase mutants, especially significant in *cfbp1* and *cyfbp cfbp1*, underlying the dramatic change in their phenotypes. The inactivation of cyFBP leads to an overall accumulation of F1,6BP, hexose-phosphates, and thus of TPs during the light period, resulting in the increase in the starch level. However, subcellular metabolite analysis will be required to confirm this interpretation (Geigenberger *et al.*, 2011). The amounts of most of the sugars increased, mainly maltose and isomaltose, the main products of starch mobilization in the chloroplast, while the sucrose level was similar to that of the wild type. Cho and co-workers (2011) also showed that *Arabidopsis* plants defective in the maltose transporter (MEX1) and the plastidic glucose translocator (pGlcT) resulted in severely reduced photosynthetic activities, a decrease of sucrose content and starch turnover, and growth retardation. A remarkable increase in trehalose content was detected in the *cyfbp* mutants. In fact, some authors have proposed that trehalose-6-phosphate (Tre6P), the intermediate of trehalose synthesis, is a component in a signalling pathway that mediates the regulation of the accumulation and/or turnover of transitory starch in *Arabidopsis* leaves, potentially linking the management of these reserves to the availability and demand for sucrose (Martins *et al.*, 2013). The positive effects of trehalose include a decrease in photo-oxidative damage, as a potential protective element (Bae *et al.*, 2005). As far as the *cyfbp* phenotype was concerned, no effect was detected in amino acid biosynthesis or other metabolic pathways when *cyFBP* was lacking.

In contrast, the inactivation of *cFBP1* had a profound effect on photosynthetic carbon metabolism and photorespiration, leading in general to alterations in the redox status as revealed by changes in ascorbate levels. In addition, it also affected other pathways in the plants, such as amino acid and organic acid metabolism in mitochondria. As expected, the lack of *cFBP1* in the light led to an accumulation of F1,6BP, TPs, and 3-PGA, and to a decline in the levels of hexose-phosphates and many sugars, including sucrose, glucose, fructose, and trehalose (signalling) and maltose (starch degradation), leading to a small rosette size. On the other hand, amino acid synthesis was also affected; the serine content diminished in *cfbp1* and the double mutant, wheras the glycine level rose in *cfbp1* during the light period. Both amino acids are involved in photorespiratory and non-photorespiratory pathways, and the opposite changes in serine and glycine content indicate that, during photorespiration, glycine decarboxylase activity is altered. Timm and colleagues suggest that serine, possibly together with glycine, acts as a metabolic signal for the transcriptional regulation of photorespiration, particularly in the glycine to serine interconversion reactions (Timm *et al.*, 2013). Moreover, during an imbalance between sucrose/starch synthesis and the production of phosphorylated intermediates in the Calvin–Benson cycle, photorespiration might provide the cell with an alternative pathway for the synthesis of sink products, such as glycine and serine (Harley and Sharkey, 1991).

In addition, the inactivation of chloroplast FBPase in *cfbp1* and *cyfbp cfbp1* led to a fall in the levels of several organic acids involved in the TCA cycle in mitochondria, which may be a secondary consequence of the decrease in carbon fixation, including, l-tryptophan, l-phenylalanine, l-tyrosine, and shikimate, a precursor in aromatic amino acid biosynthesis. These aromatic amino acids can be precursors of numerous natural products in plants, such as pigments, alkaloids, hormones, and cell wall components.

Finally, an even sharper increase in F1,6BP and TP accumulation is observed in *cyfbp cfbp1* as a consequence of both cFBP1 and cyFBP inactivation and thus a decline in sucrose content. Despite displaying a similar *cfbp1* phenotype and metabolite profile, it is interesting to observe some *cyfbp* behaviours, such as the changes in starch levels, suggesting a combined inheritance in this mutant and raising the interesting question of how these plants can increase their starch content in the absence of cFBP1. Moreover, the survival of the double mutant lacking enzymes that regulate essential metabolic steps is remarkable. The slightly higher *cFBP2* gene expression in the *cyfbp cfbp1* mutant might suggest a possible compensation of the depleted carbohydrate metabolism. Thus, the redundant function of the plastidial FBPases could not be considered.

To sum up, taken together, the results of the analysis of individual and double knockout cFBP1 and cyFBP mutants lead to the suggestion that both FBPases play important roles in sucrose and starch synthesis and contribute significantly to regulating carbohydrate turnover in plants. In addition, the lack of cFBP1 induced cell structural deficiencies, and reduced plant growth. The cFBP2 isoform could not substitute the function of the other two isoforms. In addition, this study has uncovered a relationship between sugar turnover, biomass, protein content, and other important metabolic pathways, the most important being photorespiration, amino acid synthesis, and the TCA cycle.

## Supplementary data

Supplementary data are available at *JXB* online.

Figure S1. (A) Phenotype reversion in *cyfbp* and *cfbp1* mutants complemented with translationally GFP-fused FBPases cloned into the pGWB4 vector. (B) cFBP2 gene expression using semi-quantitative RT-PCR in rosette leaves of 20-day-old *Arabidopsis* plants. (C) PFP enzymatic activity.

Figure S2. (A) Maltose transporter, plastidic glucose translocator, and glucose 6-phosphate/phosphate translocator 1 and 2 gene expression using semi-quantitative RT–PCR in rosette leaves of 20-day-old *Arabidopsis* plants. (B, C) Western blot analysis using anti-cyFBP and anti-cFBP1 antibodies.

Table S1. Gene-specific oligonucleotides used for semi-quantitative PCR.

Table S2. Stomatal density and stomatal index in epidermis of leaves of the wild type and *cyfbp*, *cfbp1*, and *cyfbp cfbp1* mutants at the adaxial and abaxial surfaces.

Tables S3. Changes in *Arabidopsis* leaf metabolite levels at the end of the night in *cyfbp*, *cfbp1*, and *cyfbp cfbp1* knockout lines relative to the wild type.

Table S4. Changes in Arabidopsis leaf metabolite levels after 8h illumination in *cyfbp*, *cfbp1*, and *cyfbp cfbp1* knockout lines relative to the wild type.

Supplementary Data
