# Supplementary material for: Disruption of both chloroplastic and cytosolic FBPase genes results in a dwarf phenotype and important starch and metabolite changes in Arabidopsis thaliana
Source: J Exp Bot. 2015 Mar 5;66(9):2673–89. doi: 10.1093/jxb/erv062 (PMC4986871; doi:10.1093/jxb/erv062)
Supplement: Supplementary Data [file supp_66_9_2673__index.html]

Disruption of both chloroplastic and cytosolic FBPase genes results in a dwarf phenotype and important starch and metabolite changes in Arabidopsis thaliana — Disruption of both chloroplastic and cytosolic FBPase genes results in a dwarf phenotype and important starch and metabolite changes in Arabidopsis thaliana — Supplementary Data 

# Disruption of both chloroplastic and cytosolic FBPase genes results in a dwarf phenotype and important starch and metabolite changes in *Arabidopsis thaliana*

## Supplementary Data

Data files

**Files in this Data Supplement:**

- Supplementary Data - Supplementary Data
