# Supplementary material for: Disruption of both chloroplastic and cytosolic FBPase genes results in a dwarf phenotype and important starch and metabolite changes in Arabidopsis thaliana
Source: J Exp Bot. 2015 Mar 5;66(9):2673–89. doi: 10.1093/jxb/erv062 (PMC4986871; doi:10.1093/jxb/erv062)
Supplement: Supplementary Data [file supp_erv062_jexbot139378_file001.pdf]

Disruption of both chloroplastic and cytosolic FBPases genes results in dwarf phenotype and important starch and metabolite changes in Arabidopsis thaliana

José A. Rojas-González[1], Mauricio Soto-Súarez[1*], Ángel García-Díaz[1**], María C. Romero-Puertas[1], Luisa M. Sandalio[1], Ángel Mérida[2], Ina Thormählen[3], Peter Geigenberger[3], Antonio J. Serrato[1] and Mariam Sahrawy[1§]

Supplementary Figure S1

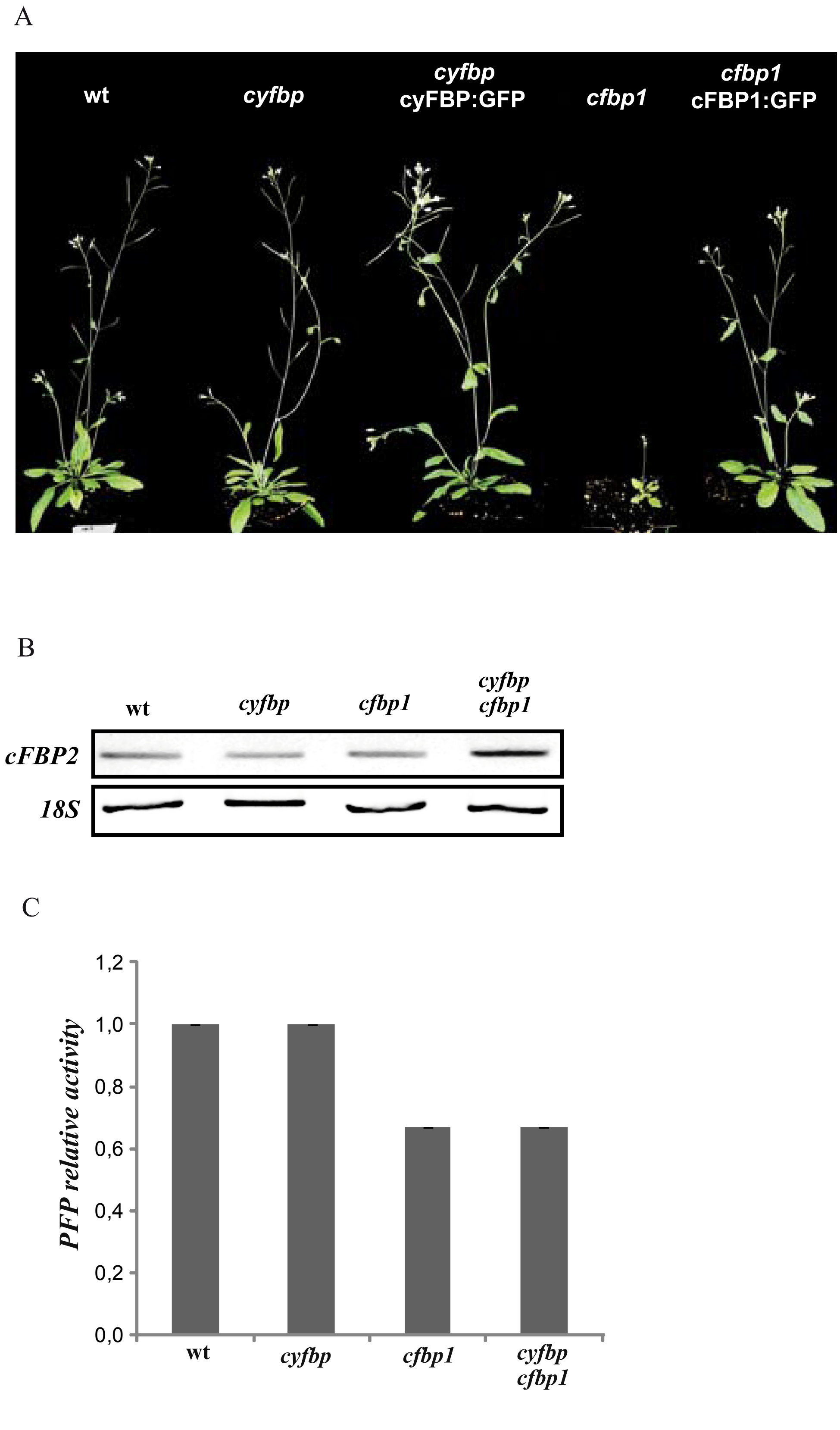

Supplementary Figure S2

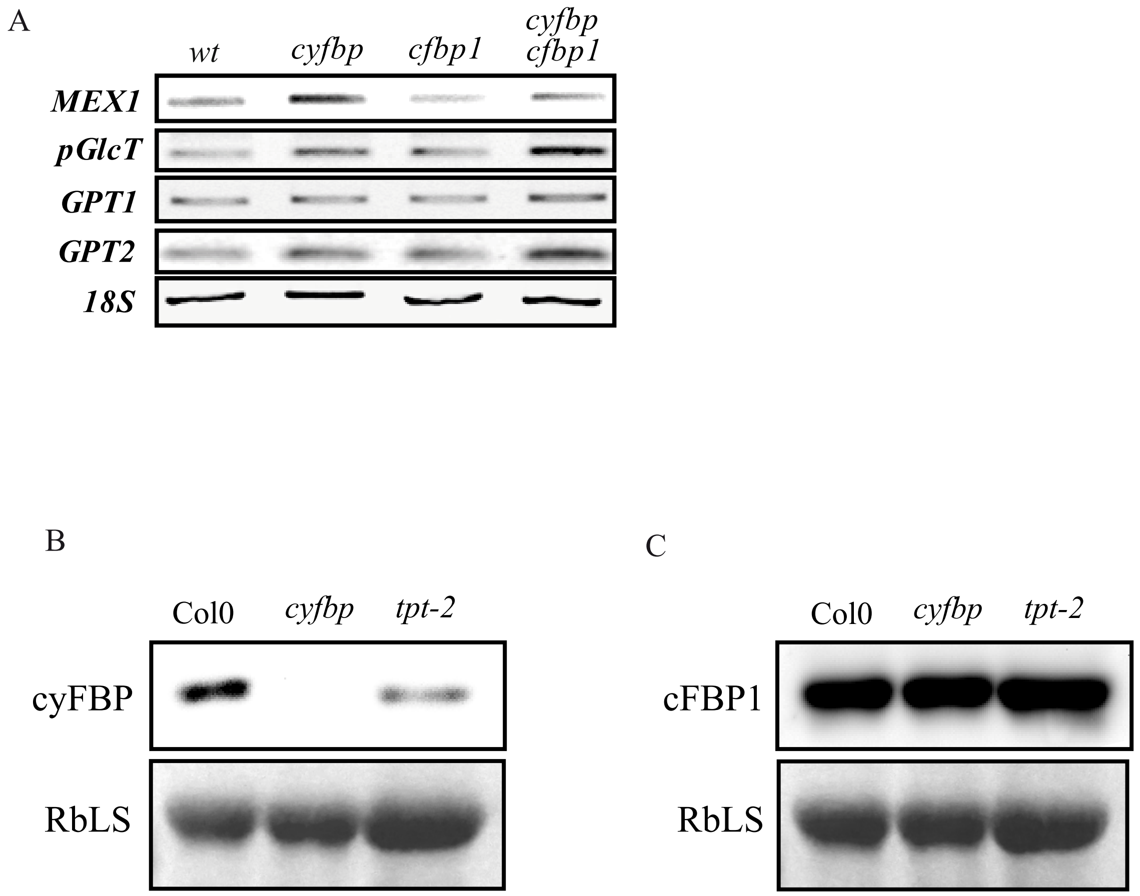

Supplementary Table S1 Gene-specific oligonucleotides used for semi-quantitative PCR

Oligos	Gene Analyzed	Primer sequence
CYFBP F	AT1G43670	GAGAGACAGAGACAGTAAG
CYFBP R		ACCCGGCTGACAAGAAAAGCCCCAA
LBSALK		TGGTTCACGTAGTGGGCCATCG
CFBP F	AT3G54050	GATATCTCAGCTCTTGGGTC
CFBP1 R		GATGTATCTCAGTCGGTTGG
GABI		CCCATTTGGACGTGAATGTAGACAC
18s RNA F	Arabidopsis	AATATACGCTATTGGAGCTGG
18s RNA R		ATGGCTCATTAAATCAGTTAT
FeSOD1 F	AT4G25100	TGCTCTTTTCTGAGTGTGTGTGCG
FeSOD1 R		TGAATAATGGGCCATGCCAAAC
FeSOD3 F	AT5G23310	CATCAGTGAGCCCTGTATGGTGAC
FeSOD3 R		ATACTCTCAGTCACGTGCGGGTC
MnSOD1 F	AT3G10920	GAGATGAACCAGTTCCAGCTCAG
MnSOD1 R		CAACGTACCACACAGCTGAGTTG
CuZnSOD1 F	AT1G08830	AACTCAGCCTGGCTACTGGAAAC
CuZnSOD1 R		CACACAACTACCAAACCCAGGTC
CuZnSOD2 F	AT2G28190	GAACAATGGTGAAGGCTGTG
CuZnSOD2 R		GTGACCACCTTTCCCAAGAT
CuZnSOD3 F	AT5G18100	AGTATTCCATACTCGGGAGGGCG
CuZnSOD3 R		GCATCCGCAGATGATTGAAGTCC
GPT1 5	AT4G24620	AAGTTCTCGCCCTGCAAAAGC
GPT1 3		CGTACAGGTCATCCACATTGC
GPT2 5	AT1G61800	AGTGGCACAAAGTGTGTTTTACC
GPT2 3		TCCTCACTGCTTCGCCTGTGAGT
pGlcT 5	AT5G16450	TTTCTCTCGGCATGCACTGG
pGlcT 3		CTATTTCCTCCAGTGATCGACC
MEX1 5	AT5G1752	TTGATGTGGCTCACTGGTTCG
MEX1 3		GTTGTGACCATAAGCCACTGC

Supplementary Table S2. Stomatal density and stomatal index in epidermis of leaves of the wild-type and *cyfbp*, *cfbp1*, and *cyfbp cfbp1* mutants at the adaxial and abaxial surfaces.

	Stomatal density		Stomatal index (%)	
	Adaxial	Abaxial	Adaxial	Abaxial
Wild-type	384±26	215±13	23±2	12±1
cyfbp	228±20 *	315±36 *	15±1 *	16±3
cfbp1	296±30 *	349±67 *	10±1 *	18±2 *
cyfbp cfbp1	274±44 *	442±41 *	11±1 *	14±3

Stomatal density, number stomata/mm^2; ± S.D. (n=8)

Stomatal index, SI = number of stomata/(number of epidermal cells + number of stomata) × 100

Data represents average ±SD for three different leaves per experiment. Asterisks (*) indicate a significant differences between means (Student's t-test, P <0.005).
(SD density of stomata=number of stomata per mm^2) and stomata index determination (SI= number of stomata/ (number of epidermal cells + number of stomata) × 100)

Supplementary Table S3. Changes in Arabidopsis leaf metabolite levels at the end of the night in *cyfbp*, *cfbp1* and *cyfbp cfbp1* knockout lines relative to the wild type. Metabolite profiling was performed using GC-TOF-MS analysis and fluorescence spectroscopy. Results are means ± SD (n = 6).Values that are significantly different from the wild type according to Student's t-test are indicated in bold (P value ≤ 0.05).

		Dark					
Metabolites	WT	cyfbp	p-value	cfbp1	p-value	cyfbp x cfbp1	p-value
Sugars							
Arabinose	1,00 ± 0,20	0,89 ± 0,50	0,623	**0,56 ± 0,19**	**0,003**	0,76 ± 0,20	0,066
Fructose	1,00 ± 0,80	0,65 ± 0,78	0,460	**0,12 ± 0,04**	**0,023**	**0,15 ± 0,05**	**0,026**
Fucose	1,00 ± 0,28	0,90 ± 0,50	0,690	**0,47 ± 0,16**	**0,002**	**0,53 ± 0,12**	**0,007**
Galactose	1,00 ± 0,60	0,83 ± 0,63	0,638	0,52 ± 0,15	0,088	0,61 ± 0,18	0,157
Glucose	1,00 ± 0,71	0,67 ± 0,57	0,391	**0,11 ± 0,07**	**0,013**	**0,07 ± 0,02**	**0,010**
Isomaltose	1,00 ± 0,07	0,67 ± 0,72	0,477	n.d.		n.d.	
Maltose	1,00 ± 0,27	1,42 ± 0,83	0,271	**0,20 ± 0,07**	**<0,001**	**0,35 ± 0,09**	**<0,001**
Mannose	1,00 ± 0,58	0,80 ± 0,58	0,559	**0,27 ± 0,08**	**0,012**	**0,33 ± 0,10**	**0,019**
Psicose	1,00 ± 0,35	1,10 ± 0,67	0,744	**0,40 ± 0,13**	**0,006**	**0,53 ± 0,16**	**0,013**
Rhamnose	1,00 ± 0,26	0,95 ± 0,63	0,865	0,85 ± 0,26	0,347	0,86 ± 0,19	0,316
Ribose	1,00 ± 0,40	0,79 ± 0,49	0,429	1,08 ± 0,46	0,766	1,11 ± 0,30	0,583
Sedoheptulose (beta-2,7-anhydro-)	1,00 ± 0,39	0,84 ± 0,53	0,556	0,61 ± 0,23	0,063	0,64 ± 0,17	0,065
Sucrose	1,00 ± 0,23	1,00 ± 0,63	0,997	**0,17 ± 0,05**	**0,000**	**0,25 ± 0,07**	**<0,001**
Trehalose	1,00 ± 0,39	1,37 ± 1,21	0,489	**0,36 ± 0,15**	**0,004**	**0,40 ± 0,13**	**0,005**
Xylose	1,00 ± 0,22	0,75 ± 0,44	0,243	**0,34 ± 0,12**	**0,000**	**0,34 ± 0,10**	**<0,001**
Phosphate esters							
Dihydroxyacetone phosphate	1,00 ± 0,10	0,98 ± 0,12	0,782	**0,42 ± 0,05**	**<0,001**	**0,42 ± 0,08**	**<0,001**
Fructose 6-phosphate	1,00 ± 0,11	**1,22 ± 0,08**	**0,003**	**0,79 ± 0,15**	**0,017**	**0,74 ± 0,14**	**0,005**
Fructose 1,6-bisphosphate			n.d.				
Glucose 1-phosphate	1,00 ± 0,24	**0,68 ± 0,22**	**0,041**	**0,69 ± 0,10**	**0,016**	0,80 ± 0,04	0,079
Glucose 6-phosphate	1,00 ± 0,09	**1,13 ± 0,04**	**0,007**	**0,64 ± 0,06**	**<0,001**	**0,65 ± 0,07**	**<0,001**
Glyceraldehyde 3-phosphate	1,00 ± 0,20	**1,30 ± 0,20**	**0,034**	0,89 ± 0,37	0,548	**2,76 ± 1,09**	**0,004**
Glycerol 3-phosphate	1,00 ± 0,28	**0,60 ± 0,29**	**0,035**	0,85 ± 0,31	0,407	**0,66 ± 0,23**	**0,046**
3-Phosphoglycerate	1,00 ± 0,16	**1,27 ± 0,21**	**0,031**	**0,51 ± 0,07**	**<0,001**	**0,62 ± 0,07**	**<0,001**
Organic acids							
Benzoate	1,00 ± 0,29	0,98 ± 0,58	0,955	0,93 ± 0,19	0,640	0,84 ± 0,24	0,334
Butanoate (4-amino-)	1,00 ± 0,65	0,67 ± 0,64	0,394	1,34 ± 0,74	0,418	**2,01 ± 0,48**	**0,012**
Fumarate	1,00 ± 0,26	0,77 ± 0,44	0,303	**0,28 ± 0,12**	**<0,001**	**0,13 ± 0,04**	**<0,001**
Galactonate	1,00 ± 0,22	0,88 ± 0,50	0,616	0,86 ± 0,36	0,427	**0,70 ± 0,20**	**0,034**
Gluconate	1,00 ± 1,41	0,07 ± 0,04	0,137	0,10 ± 0,02	0,191	0,06 ± 0,01	0,306

Glutarate (2-oxo-)	1,00 ± 0,32	1,16 ± 0,68	0,609	0,96 ± 0,21	0,817	0,94 ± 0,23	0,732
Glycerate	1,00 ± 0,30	0,82 ± 0,46	0,447	**0,50 ± 0,10**	**0,003**	**0,37 ± 0,11**	**<0,001**
Gulonate	1,00 ± 0,92	0,91 ± 1,03	0,881	**0,12 ± 0,02**	**0,042**	**0,09 ± 0,03**	**0,037**
Hexadecanoate	1,00 ± 0,40	1,31 ± 1,17	0,548	1,07 ± 0,45	0,797	0,90 ± 0,25	0,631
Malate	1,00 ± 0,32	0,87 ± 0,55	0,640	**0,25 ± 0,12**	**<0,001**	**0,15 ± 0,04**	**<0,001**
Maleate	1,00 ± 0,37	0,95 ± 0,55	0,853	0,83 ± 0,39	0,465	**0,54 ± 0,21**	**0,025**
Nicotinate	1,00 ± 0,31	0,87 ± 0,48	0,586	0,76 ± 0,23	0,150	0,94 ± 0,27	0,726
Octadecanoate	1,00 ± 0,45	1,14 ± 1,09	0,773	1,05 ± 0,46	0,856	0,86 ± 0,25	0,522
Pyruvate	1,00 ± 0,35	1,04 ± 0,69	0,910	1,08 ± 0,29	0,658	0,84 ± 0,24	0,376
Quinate	1,00 ± 0,35	0,88 ± 0,55	0,750	1,18 ± 0,36	0,505	1,26 ± 0,37	0,350
Shikimate	1,00 ± 0,27	1,02 ± 0,55	0,942	0,77 ± 0,25	0,155	0,84 ± 0,21	0,266
Sinapate (cis-)	1,00 ± 0,40	0,78 ± 0,43	0,374	0,83 ± 0,34	0,449	0,71 ± 0,27	0,161
Sinapate (trans-)	1,00 ± 0,40	0,73 ± 0,38	0,256	0,86 ± 0,33	0,528	0,69 ± 0,27	0,157
Succinate	1,00 ± 0,36	1,39 ± 0,93	0,361	**0,35 ± 0,13**	**0,002**	**0,30 ± 0,07**	**<0,001**
Threonate	1,00 ± 0,20	0,77 ± 0,42	0,264	**0,51 ± 0,18**	**0,001**	**0,35 ± 0,11**	**<0,001**
Amino acids							
Alanine	1,00 ± 0,38	0,78 ± 0,50	0,409	1,09 ± 0,40	0,703	**2,01 ± 0,35**	**<0,001**
Arginine				n.d.			
Aspartate	1,00 ± 0,50	0,92 ± 0,60	0,803	**3,61 ± 2,30**	**0,022**	**3,64 ± 0,99**	**<0,001**
Glutamate	1,00 ± 0,96	0,77 ± 0,67	0,647	2,92 ± 2,03	0,063	2,03 ± 0,76	0,066
Glycine	1,00 ± 1,22	0,46 ± 0,37	0,320	0,56 ± 0,22	0,404	0,70 ± 0,23	0,564
Isoleucine	1,00 ± 1,17	0,54 ± 0,43	0,391	1,20 ± 0,37	0,692	0,83 ± 0,09	0,726
Leucine	1,00 ± 1,08	0,60 ± 0,49	0,433	0,65 ± 0,26	0,464	0,70 ± 0,10	0,509
Proline	1,00 ± 1,86	0,26 ± 0,32	0,356	0,28 ± 0,07	0,367	0,30 ± 0,05	0,379
Pyroglutamate	1,00 ± 0,21	0,88 ± 0,40	0,538	1,05 ± 0,44	0,803	1,27 ± 0,30	0,093
Serine	1,00 ± 0,46	1,01 ± 0,82	0,986	0,55 ± 0,18	0,050	1,16 ± 0,33	0,500
Threonine	1,00 ± 0,41	0,78 ± 0,44	0,396	**16,00 ± 6,45**	**<0,001**	**8,56 ± 1,51**	**<0,001**
Tryptophan	1,00 ± 0,21	0,88 ± 0,64	0,763	0,91 ± 0,25	0,622	1,04 ± 0,23	0,840
Valine	1,00 ± 0,61	0,90 ± 0,53	0,768	0,93 ± 0,24	0,802	1,14 ± 0,25	0,604
Sugar alcohols							
Erythritol	1,00 ± 0,39	0,97 ± 0,54	0,922	0,88 ± 0,19	0,504	0,82 ± 0,24	0,355
Glycerol	1,00 ± 0,26	0,88 ± 0,50	0,603	**0,65 ± 0,17**	**0,021**	**0,65 ± 0,16**	**0,018**
Inositol (myo-)	1,00 ± 0,34	1,09 ± 0,62	0,772	**0,20 ± 0,05**	**<0,001**	**0,20 ± 0,05**	**<0,001**
Maltitol	1,00 ± 0,87	0,85 ± 0,73	0,757	**0,08 ± 0,03**	**0,027**	**0,06 ± 0,03**	**0,040**
Mannitol	1,00 ± 0,73	0,67 ± 0,36	0,346	0,77 ± 0,44	0,524	1,21 ± 0,39	0,669
Others							

Ascorbate	1,00 ± 0,89	1,76 ± 1,12	0,220	**3,14 ± 1,60**	**0,017**	**5,48 ± 1,66**	**<0,001**
Dehydroascorbate dimer	1,00 ± 0,58	1,28 ± 0,72	0,475	0,80 ± 0,25	0,451	0,89 ± 0,32	0,698
Ethanolamine	1,00 ± 0,68	0,96 ± 1,13	0,941	1,11 ± 0,63	0,774	**2,99 ± 1,54**	**0,016**
Phosphate	1,00 ± 0,21	0,75 ± 0,40	0,198	0,90 ± 0,32	0,524	1,14 ± 0,29	0,349
Spermidine	1,00 ± 0,36	0,74 ± 0,44	0,317	0,86 ± 0,30	0,496	0,69 ± 0,31	0,174
Uracil	1,00 ± 0,32	1,12 ± 0,84	0,745	1,16 ± 0,32	0,410	0,93 ± 0,25	0,681

n.d.: not detectable

Supplementary Table S4. Changes in Arabidopsis leaf metabolite levels after 8 h illumination (16 h light/8 h dark) in *cyfbp*, *cfbp1,* and *cyfbp cfbp1* knockout lines compared relative to the wild type. Metabolite profiling was performed using GC-TOF-MS analysis and fluorescence spectroscopy. Results are means ± SD (n = 6).Values that are significantly different from the wild type according to Student's t-test are indicated in bold (P value ≤ 0.05).

			Light				
Metabolites	WT	cyfbp	p-value	cfbp1	p-value	cyfbp x cfbp1	p-value
Sugars							
Arabinose	1,00 ± 0,43	1,48 ± 1,17	0,369	**0,58 ± 0,10**	**0,042**	**0,56 ± 0,20**	**0,047**
Fructose	1,00 ± 0,83	1,54 ± 1,50	0,458	**0,13 ± 0,03**	**0,028**	**0,10 ± 0,06**	**0,024**
Fucose	1,00 ± 0,40	1,45 ± 1,15	0,381	**0,50 ± 0,11**	**0,015**	**0,40 ± 0,15**	**0,006**
Galactose	1,00 ± 0,57	2,71 ± 2,15	0,090	0,48 ± 0,19	0,059	0,52 ± 0,21	0,080
Glucose	1,00 ± 0,68	1,74 ± 1,72	0,348	**0,11 ± 0,04**	**0,010**	**0,07 ± 0,04**	**0,008**
Isomaltose	1,00 ± 0,53	**4,51 ± 3,54**	**0,037**	0,47 ± 0,10	0,090	**0,29 ± 0,09**	**0,033**
Maltose	1,00 ± 0,42	3,93 ± 4,27	0,126	**0,22 ± 0,08**	**0,018**	**0,21 ± 0,07**	**0,001**
Mannose	1,00 ± 0,59	1,63 ± 1,30	0,307	**0,27 ± 0,08**	**0,013**	**0,25 ± 0,10**	**0,011**
Psicose	1,00 ± 0,36	2,33 ± 1,82	0,110	**0,28 ± 0,10**	**<0,001**	**0,34 ± 0,11**	**0,002**
Rhamnose	1,00 ± 0,43	1,66 ± 1,35	0,277	0,79 ± 0,16	0,277	0,67 ± 0,21	0,118
Ribose	1,00 ± 0,36	1,77 ± 1,51	0,256	1,18 ± 0,34	0,390	1,22 ± 0,44	0,370
Sedoheptulose (beta-2,7-anhydro-)	1,00 ± 0,43	1,31 ± 1,04	0,512	**0,47 ± 0,10**	**0,014**	**0,36 ± 0,13**	**0,005**
Sucrose	1,00 ± 0,43	2,21 ± 2,01	0,179	**0,55 ± 0,13**	**0,034**	**0,27 ± 0,07**	**0,002**
Trehalose	1,00 ± 0,44	2,37 ± 2,31	0,182	**0,31 ± 0,11**	**0,004**	**0,31 ± 0,17**	**0,005**
Xylose	1,00 ± 0,48	1,40 ± 1,05	0,421	**0,35 ± 0,08**	**0,009**	**0,30 ± 0,10**	**0,006**
Phosphate ester							
Dihydroxyacetone phosphate	1,00 ± 0,05	**1,26 ± 0,14**	**0,002**	**1,65 ± 0,55**	**0,017**	**3,08 ± 0,29**	**<0,001**
Fructose 6-phosphate	1,00 ± 0,08	**1,23 ± 0,13**	**0,004**	0,97 ± 0,11	0,631	0,99 ± 0,10	0,887
Fructose 1,6-bisphosphate	1,00 ± 0,38	**4,61 ± 2,00**	**0,002**	**16,54 ± 11,60**	**0,009**	**59,76 ± 7,22**	**<0,001**
Glucose 1-phosphate	1,00 ± 0,27	1,06 ± 0,43	0,776	1,05 ± 0,21	0,741	0,74 ± 0,16	0,065
Glucose 6-phosphate	1,00 ± 0,06	**1,25 ± 0,06**	**<0,001**	0,95 ± 0,05	0,108	**0,91 ± 0,06**	**0,033**
Glyceraldehyde 3-phosphate	1,00 ± 0,33	**4,83 ± 1,13**	**<0,001**	**12,54 ± 5,20**	**<0,001**	**29,77 ± 3,92**	**<0,001**
Glycerol 3-phosphate	1,00 ± 0,42	0,74 ± 0,50	0,359	0,63 ± 0,30	0,111	**0,45 ± 0,20**	**0,017**
3-Phosphoglycerate	1,00 ± 0,09	**2,88 ± 0,24**	**<0,001**	**1,85 ± 0,54**	**0,004**	**2,27 ± 0,15**	**<0,001**
Organic acids							
Benzoate	1,00 ± 0,37	1,47 ± 1,26	0,399	**0,60 ± 0,18**	**0,039**	**0,57 ± 0,22**	**0,036**
Butanoate (4-amino-)	1,00 ± 0,57	1,17 ± 1,20	0,760	**2,86 ± 1,65**	**0,026**	2,27 ± 1,63	0,102
Fumarate	1,00 ± 0,40	1,18 ± 0,95	0,678	**0,29 ± 0,09**	**0,002**	**0,14 ± 0,05**	**<0,001**
Galactonate	1,00 ± 0,44	1,23 ± 0,91	0,599	**0,49 ± 0,18**	**0,026**	**0,37 ± 0,12**	**0,007**
Gluconate	1,00 ± 0,29	**2,01 ± 1,04**	**0,044**	**0,29 ± 0,10**	**0,002**	**0,28 ± 0,14**	**<0,001**

Glutarate (2-oxo-)	1,00 ± 0,48	1,71 ± 1,64	0,334	0,65 ± 0,31	0,158	**0,51 ± 0,24**	**0,049**
Glycerate	1,00 ± 0,40	1,80 ± 1,17	0,146	**0,24 ± 0,04**	**<0,001**	**0,26 ± 0,10**	**0,001**
Gulonate	1,00 ± 0,37	**2,87 ± 1,17**	**0,004**	**0,06 ± 0,02**	**<0,001**	**0,04 ± 0,02**	**<0,001**
Hexadecanoate	1,00 ± 0,46	3,24 ± 4,00	0,204	0,76 ± 0,42	0,361	0,58 ± 0,39	0,117
Malate	1,00 ± 0,47	0,71 ± 0,46	0,302	**0,29 ± 0,15**	**0,005**	**0,14 ± 0,10**	**0,001**
Maleate	1,00 ± 0,31	0,91 ± 0,53	0,729	**0,49 ± 0,25**	**0,010**	**0,29 ± 0,11**	**<0,001**
Nicotinate	1,00 ± 0,34	1,04 ± 0,68	0,906	**0,49 ± 0,15**	**0,007**	**0,58 ± 0,20**	**0,026**
Octadecanoate	1,00 ± 0,48	3,24 ± 4,19	0,222	0,80 ± 0,50	0,487	0,56 ± 0,39	0,111
Pyruvate	1,00 ± 0,37	1,36 ± 1,06	0,453	0,69 ± 0,26	0,124	0,60 ± 0,27	0,061
Quinate	1,00 ± 0,59	1,47 ± 1,25	0,421	0,91 ± 0,44	0,759	1,08 ± 1,10	0,884
Shikimate	1,00 ± 0,39	1,40 ± 1,00	0,378	**0,62 ± 0,12**	**0,044**	**0,49 ± 0,16**	**0,014**
Sinapate (cis-)	1,00 ± 0,31	1,09 ± 0,65	0,760	**0,52 ± 0,10**	**0,005**	**0,37 ± 0,13**	**<0,001**
Sinapate (trans-)	1,00 ± 0,40	1,02 ± 0,74	0,953	**0,53 ± 0,20**	**0,028**	**0,39 ± 0,13**	**0,006**
Succinate	1,00 ± 0,35	1,56 ± 0,75	0,127	**0,18 ± 0,05**	**<0,001**	**0,11 ± 0,04**	**<0,001**
Threonate	1,00 ± 0,44	1,11 ± 0,80	0,776	**0,30 ± 0,09**	**0,003**	**0,22 ± 0,08**	**0,002**
<u>Amino acids</u>							
Alanine	1,00 ± 0,43	1,43 ± 1,27	0,453	1,16 ± 0,43	0,525	1,22 ± 0,53	0,445
Arginine	1,00 ± 0,48	n.d.		0,76 ± 0,08	0,243	0,83 ± 0,59	0,651
Aspartate	1,00 ± 1,10	0,61 ± 0,58	0,466	0,78 ± 0,35	0,645	0,63 ± 0,41	0,462
Glutamate	1,00 ± 0,84	4,16 ± 4,49	0,122	2,38 ± 2,55	0,236	1,20 ± 0,94	0,699
Glycine	1,00 ± 0,23	1,10 ± 0,97	0,829	**3,25 ± 2,01**	**0,036**	1,81 ± 1,23	0,184
Isoleucine	1,00 ± 0,26	1,67 ± 1,67	0,357	1,21 ± 0,14	0,110	1,03 ± 0,29	0,874
Leucine	1,00 ± 0,31	1,80 ± 1,75	0,295	**0,59 ± 0,12**	**0,012**	**0,57 ± 0,22**	**0,019**
Proline	1,00 ± 0,36	2,27 ± 3,03	0,332	**1,50 ± 0,29**	**0,026**	1,79 ± 1,17	0,148
Pyroglutamate	1,00 ± 0,37	1,00 ± 0,64	0,993	0,81 ± 0,28	0,332	0,61 ± 0,22	0,052
Serine	1,00 ± 0,43	0,88 ± 0,37	0,631	**0,50 ± 0,18**	**0,024**	**0,40 ± 0,09**	**0,008**
Threonine	1,00 ± 0,42	0,75 ± 0,34	0,271	**8,50 ± 2,06**	**<0,001**	**4,74 ± 1,43**	**<0,001**
Tryptophan	1,00 ± 0,55	1,98 ± 1,86	0,247	0,72 ± 0,28	0,292	0,57 ± 0,36	0,166
Valine	1,00 ± 0,38	1,59 ± 1,48	0,363	**0,50 ± 0,12**	**0,012**	0,68 ± 0,20	0,104
<u>Sugar alcohols</u>							
Erythritol	1,00 ± 0,42	1,16 ± 0,97	0,713	**0,54 ± 0,21**	**0,037**	**0,49 ± 0,24**	**0,041**
Glycerol	1,00 ± 0,39	1,72 ± 1,41	0,256	**0,48 ± 0,11**	**0,010**	**0,50 ± 0,25**	**0,022**
Inositol (myo-)	1,00 ± 0,47	1,57 ± 1,34	0,348	**0,13 ± 0,05**	**0,001**	**0,14 ± 0,05**	**0,001**
Maltitol	1,00 ± 0,47	5,00 ± 4,43	0,052	**0,06 ± 0,03**	**<0,001**	**0,03 ± 0,02**	**<0,001**
Mannitol	1,00 ± 0,92	1,19 ± 1,12	0,772	0,64 ± 0,28	0,423	0,51 ± 0,25	0,281
<u>Others</u>							

Ascorbate	1,00 ± 0,70	0,70 ± 0,52	0,422	1,15 ± 0,60	0,693	1,29 ± 0,80	0,525
Dehydroascorbate dimer	1,00 ± 0,56	3,71 ± 3,43	0,086	0,60 ± 0,24	0,136	0,48 ± 0,18	0,054
Ethanolamine	1,00 ± 0,68	0,96 ± 1,19	0,950	1,90 ± 1,47	0,205	**4,38 ± 2,63**	**0,012**
Phosphate	1,00 ± 0,45	1,41 ± 1,12	0,424	0,67 ± 0,29	0,162	0,61 ± 0,35	0,125
Spermidine	1,00 ± 0,42	1,10 ± 0,80	0,784	**0,55 ± 0,22**	**0,044**	**0,42 ± 0,19**	**0,012**
Uracil	1,00 ± 0,46	1,16 ± 0,85	0,702	0,66 ± 0,26	0,150	0,64 ± 0,31	0,139

n.d.: not detectable
